# Supplementary figures and images for: Sensitivity of Kβ mainline X-ray emission to structural dynamics in iron photosensitizer
Source: Phys Chem Chem Phys. 2023 Mar 30;25(15):10447–59. doi: 10.1039/d2cp05671b (PMC10091363; doi:10.1039/d2cp05671b)

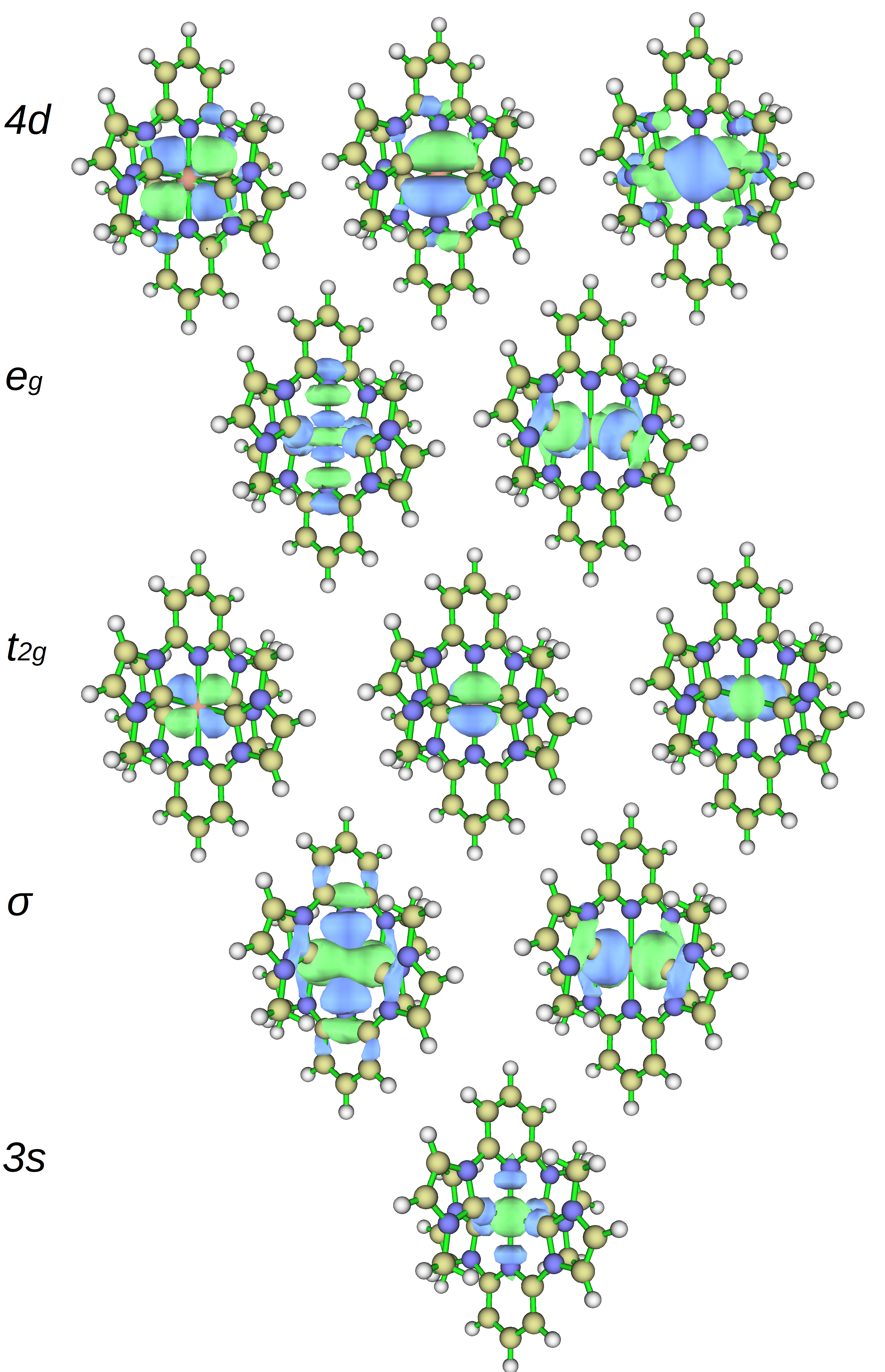

Supplement: CP-025-D2CP05671B-s001 [file CP-025-D2CP05671B-s001.zip › SI-figures/FigS1_RAS2_4.png]

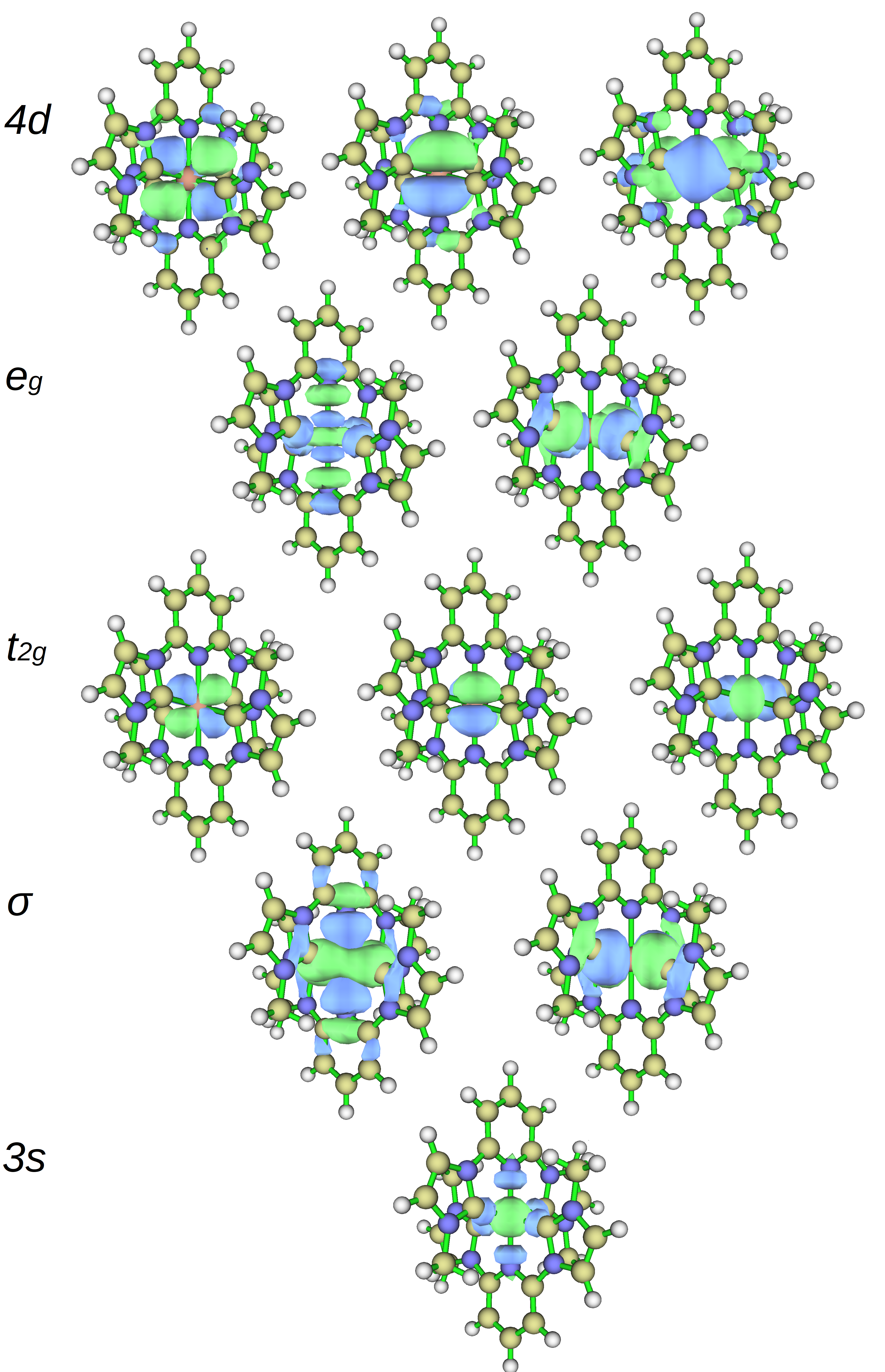

Supplement: CP-025-D2CP05671B-s001 [file CP-025-D2CP05671B-s001.zip › SI-figures/FigS1_RAS2_4.tif]

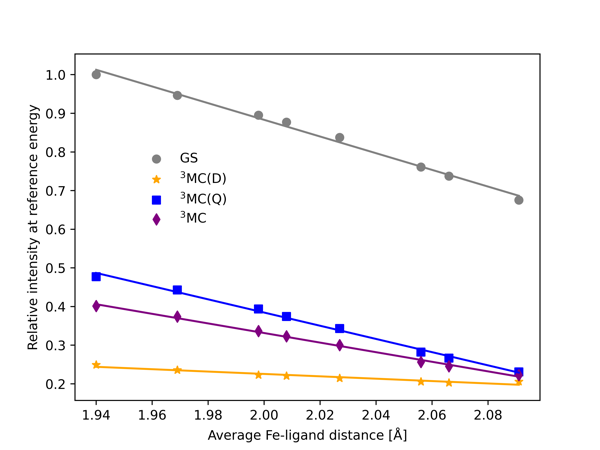

Supplement: CP-025-D2CP05671B-s001 [file CP-025-D2CP05671B-s001.zip › SI-figures/FigS10_dIdr_RAS_DQ.png]

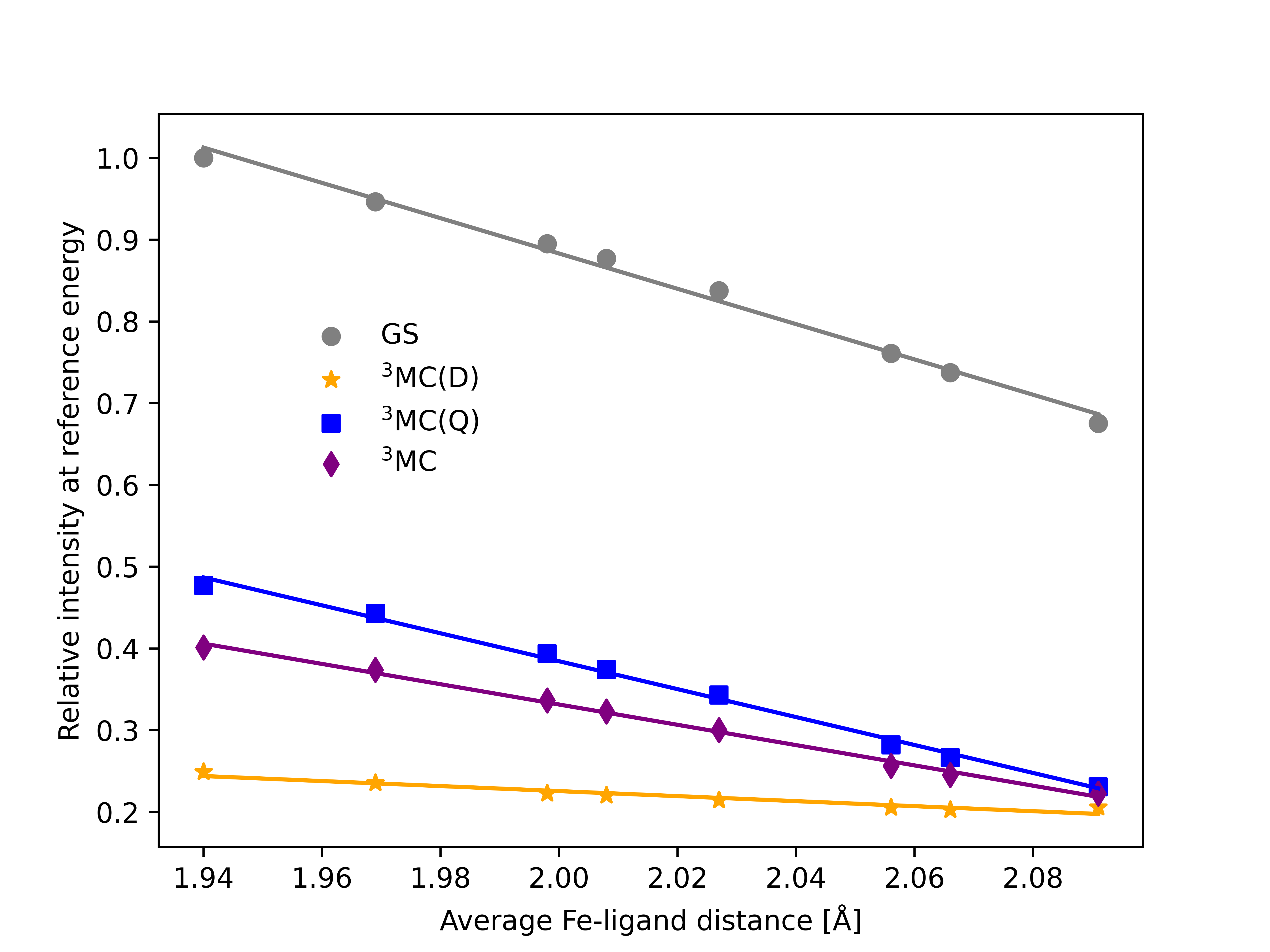

Supplement: CP-025-D2CP05671B-s001 [file CP-025-D2CP05671B-s001.zip › SI-figures/FigS10_dIdr_RAS_DQ.tif]

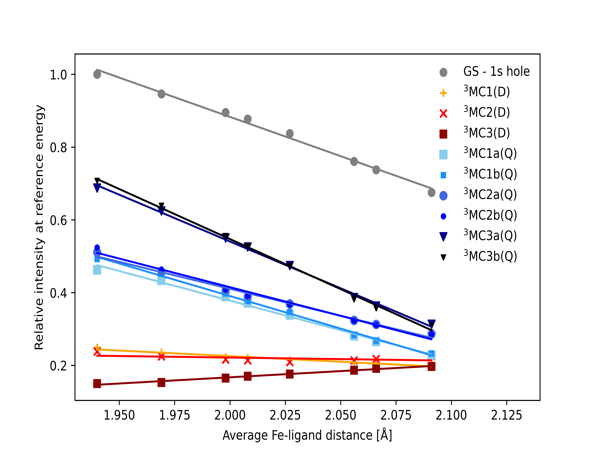

Supplement: CP-025-D2CP05671B-s001 [file CP-025-D2CP05671B-s001.zip › SI-figures/FigS11_dIdr_RAS_DQ_all.png]

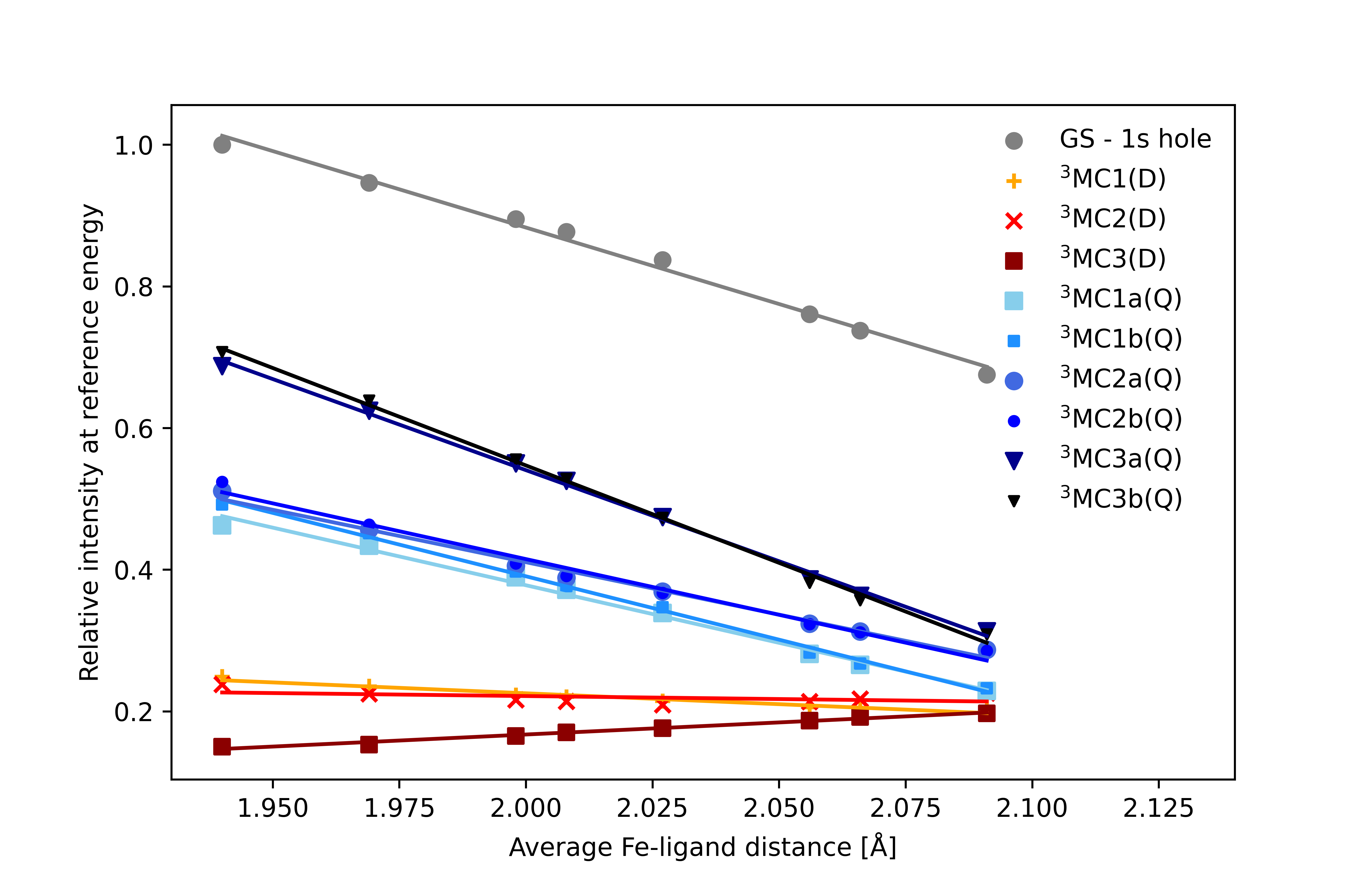

Supplement: CP-025-D2CP05671B-s001 [file CP-025-D2CP05671B-s001.zip › SI-figures/FigS11_dIdr_RAS_DQ_all.tif]

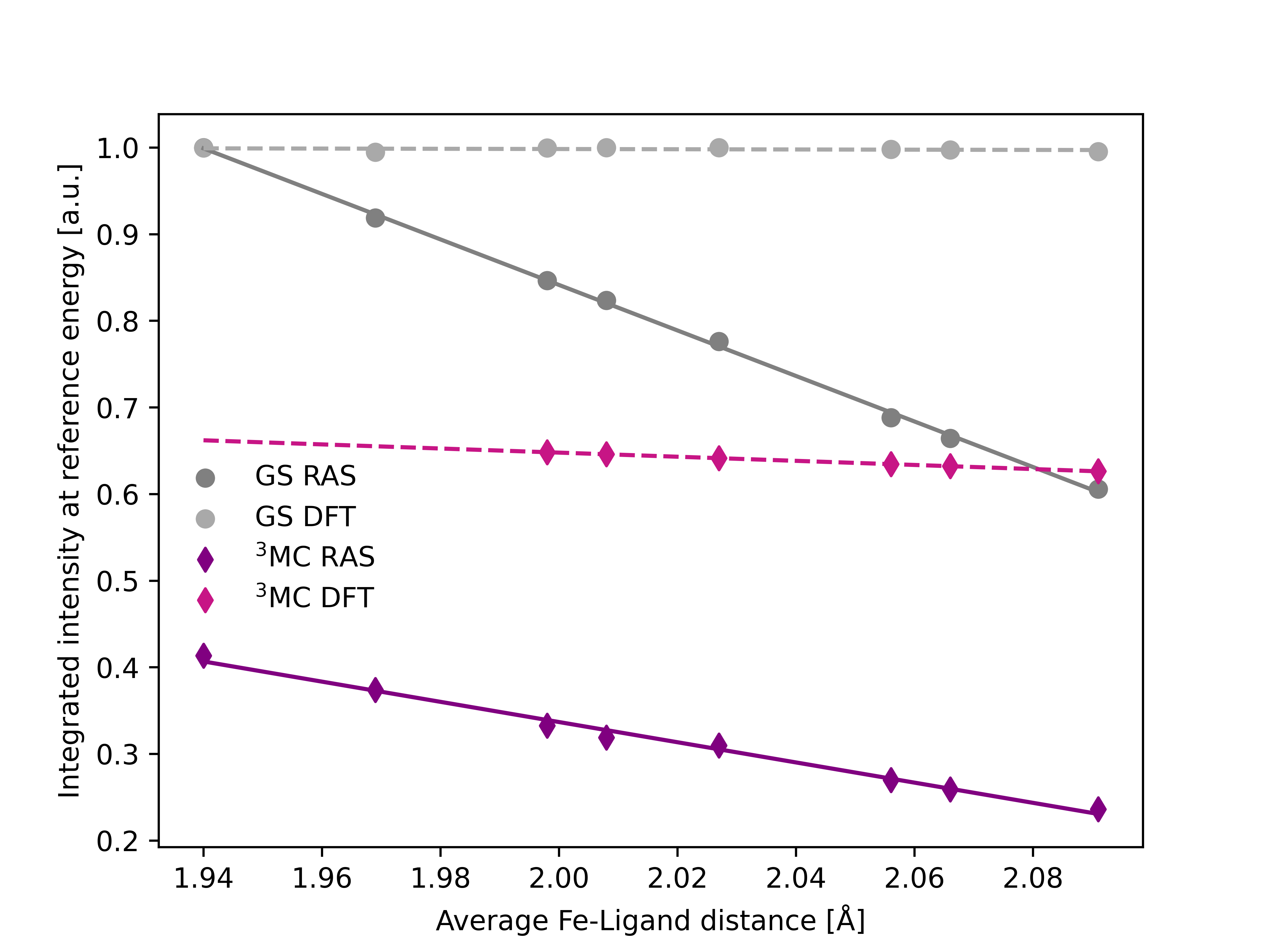

Supplement: CP-025-D2CP05671B-s001 [file CP-025-D2CP05671B-s001.zip › SI-figures/FigS12_dIdr_DFT_RAS_integrated.png]

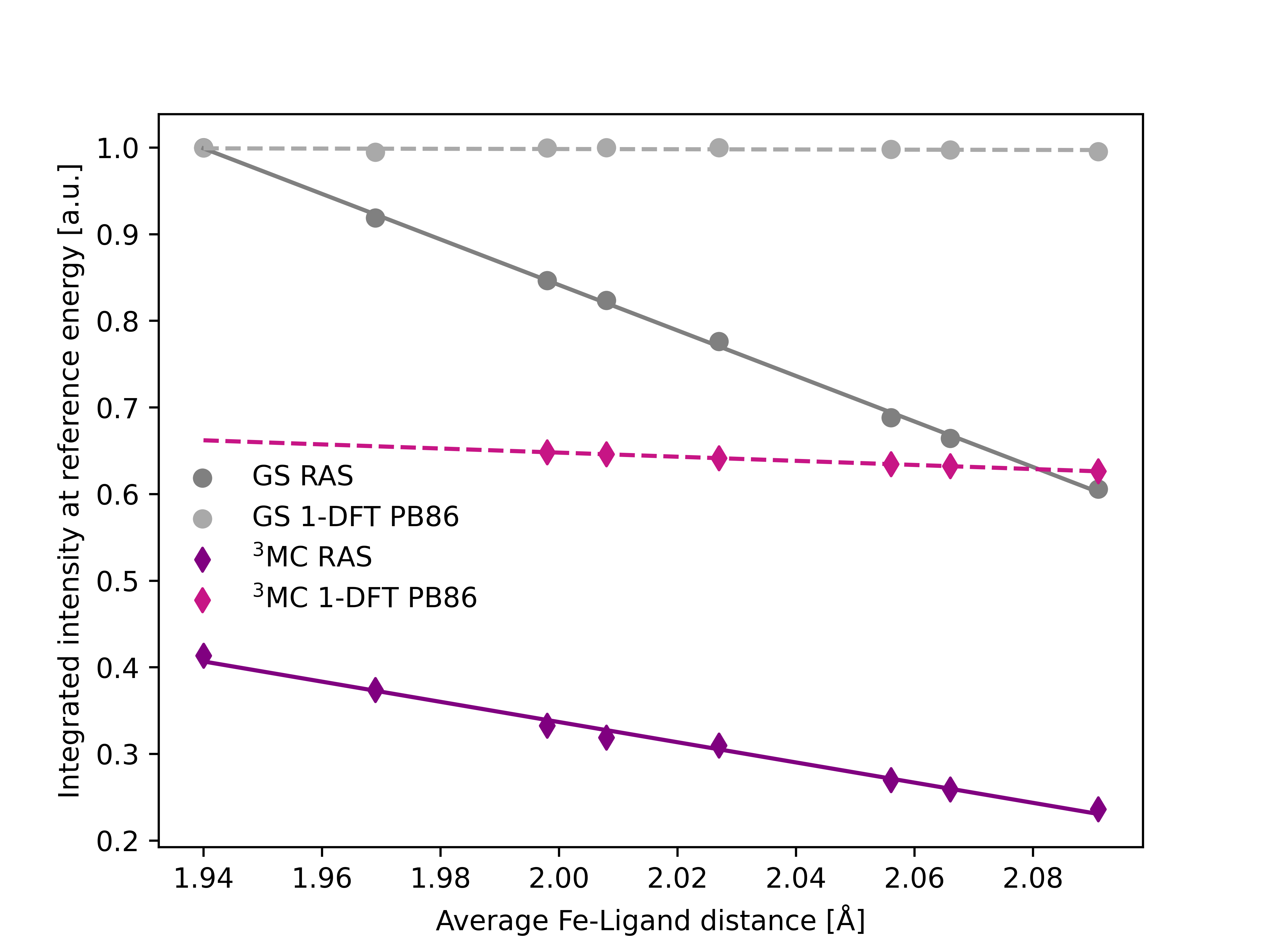

Supplement: CP-025-D2CP05671B-s001 [file CP-025-D2CP05671B-s001.zip › SI-figures/FigS12_dIdr_DFT_RAS_integrated.tif]

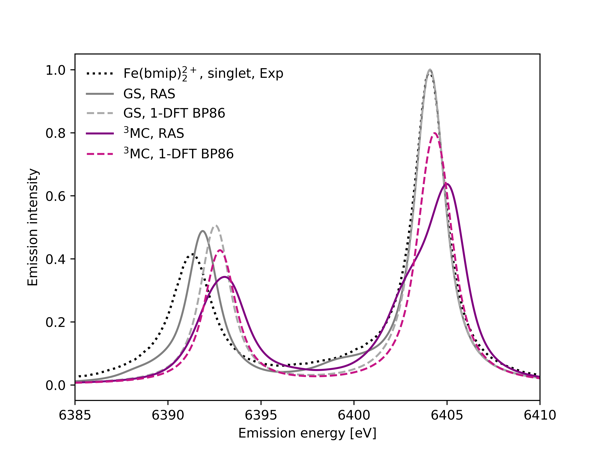

Supplement: CP-025-D2CP05671B-s001 [file CP-025-D2CP05671B-s001.zip › SI-figures/FigS13_Exp_calc_GS_3MC_spectra_Kalpha_noGeo.png]

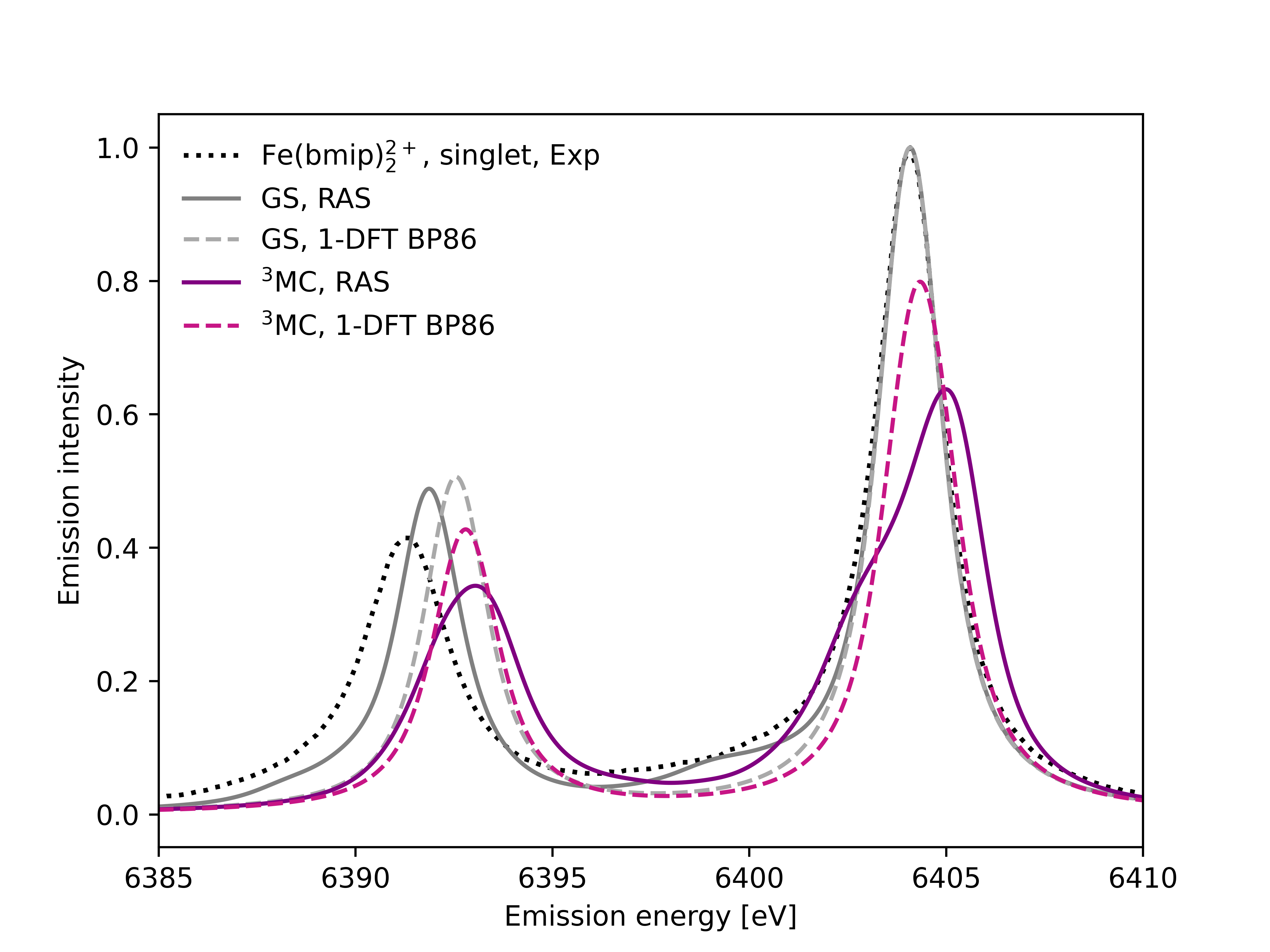

Supplement: CP-025-D2CP05671B-s001 [file CP-025-D2CP05671B-s001.zip › SI-figures/FigS13_Exp_calc_GS_3MC_spectra_Kalpha_noGeo.tif]

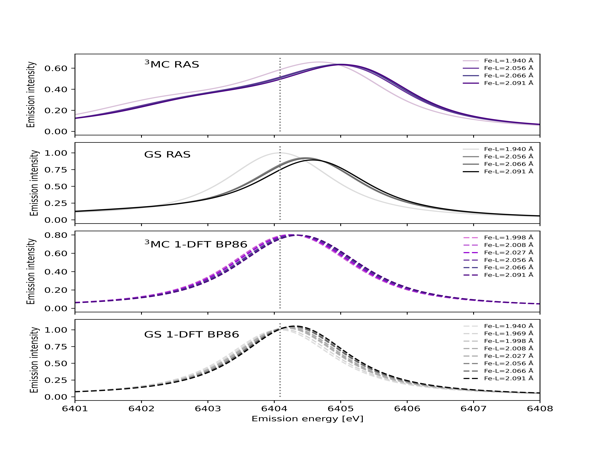

Supplement: CP-025-D2CP05671B-s001 [file CP-025-D2CP05671B-s001.zip › SI-figures/FigS14_Kalpha_GS_3MC1_all_separate.png]

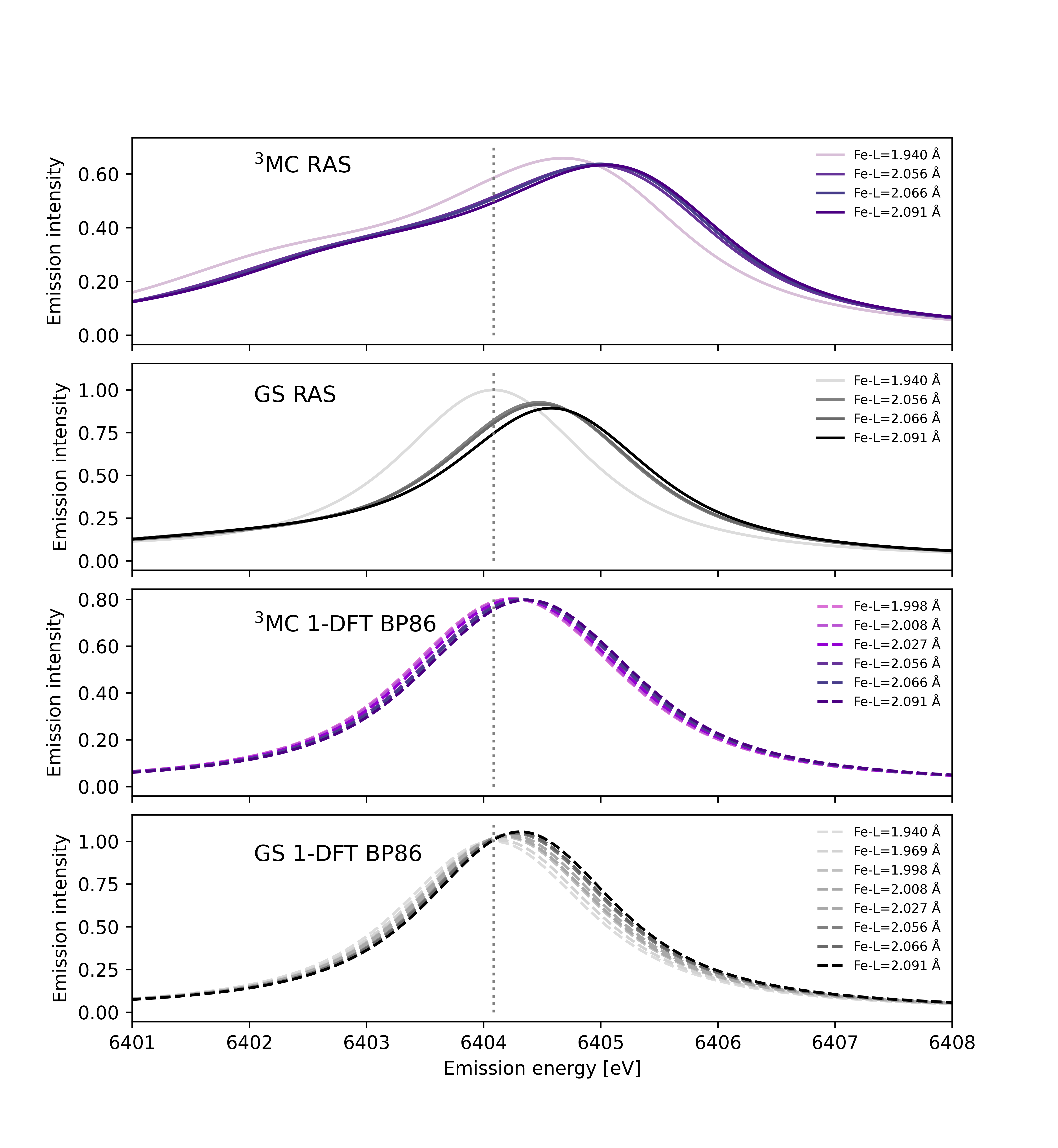

Supplement: CP-025-D2CP05671B-s001 [file CP-025-D2CP05671B-s001.zip › SI-figures/FigS14_Kalpha_GS_3MC1_all_separate.tif]

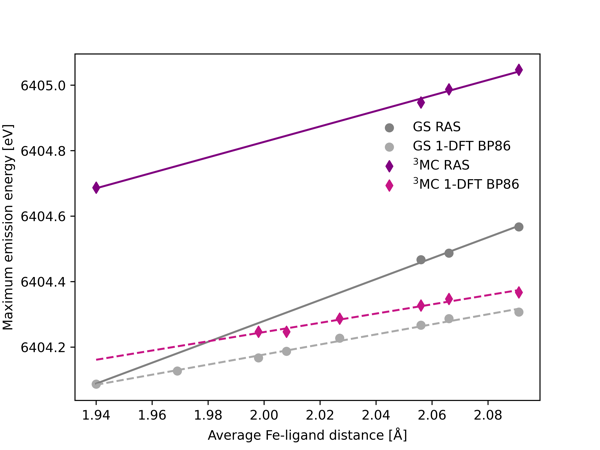

Supplement: CP-025-D2CP05671B-s001 [file CP-025-D2CP05671B-s001.zip › SI-figures/FigS15_dEdr_DFT_RAS_6_points_Kalpha.png]

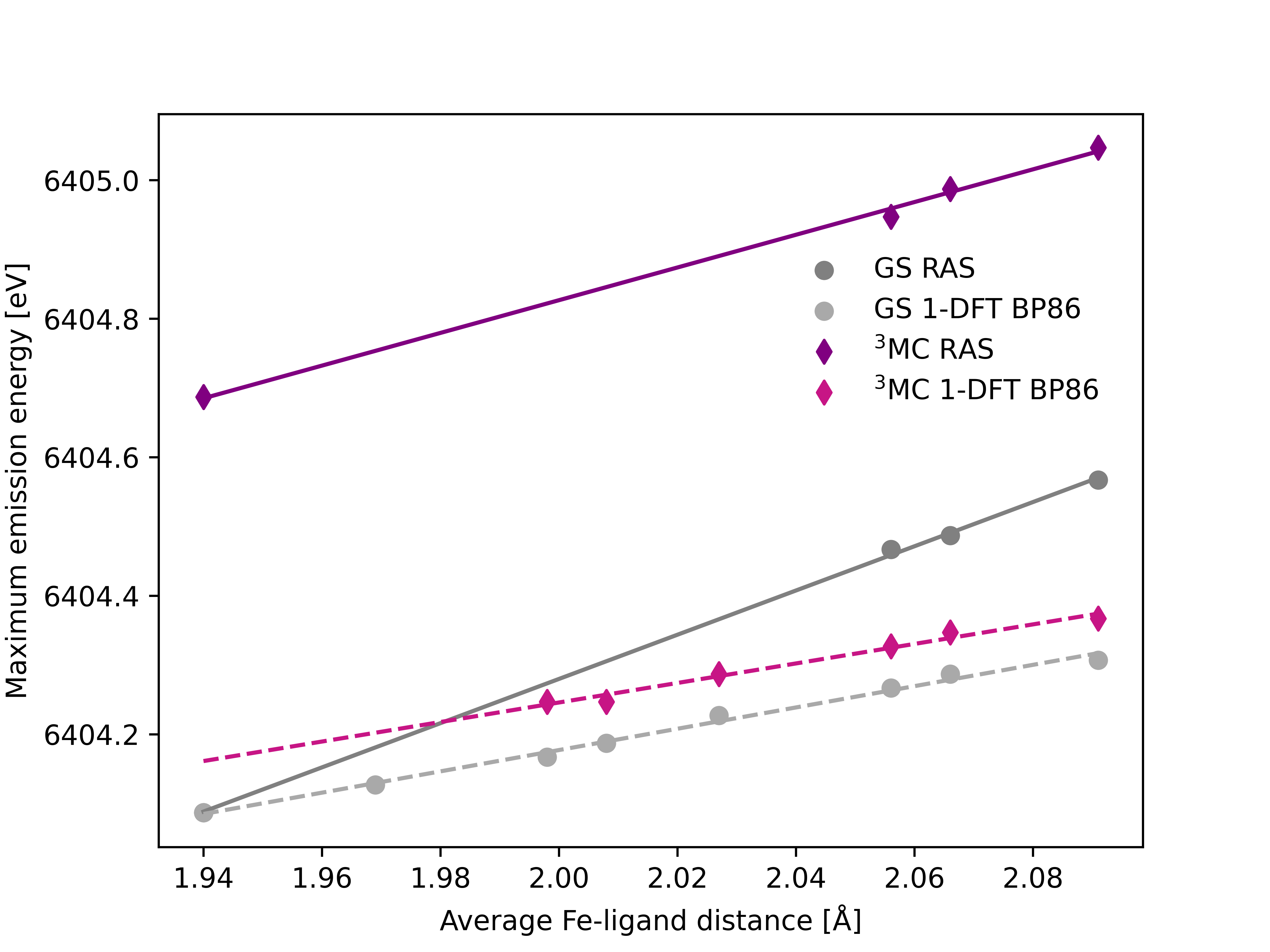

Supplement: CP-025-D2CP05671B-s001 [file CP-025-D2CP05671B-s001.zip › SI-figures/FigS15_dEdr_DFT_RAS_6_points_Kalpha.tif]

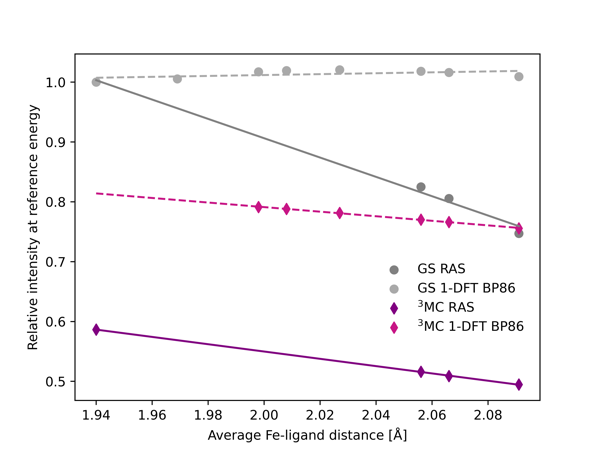

Supplement: CP-025-D2CP05671B-s001 [file CP-025-D2CP05671B-s001.zip › SI-figures/FigS16_dIdr_DFT_RAS_6_points_use_Kalpha.png]

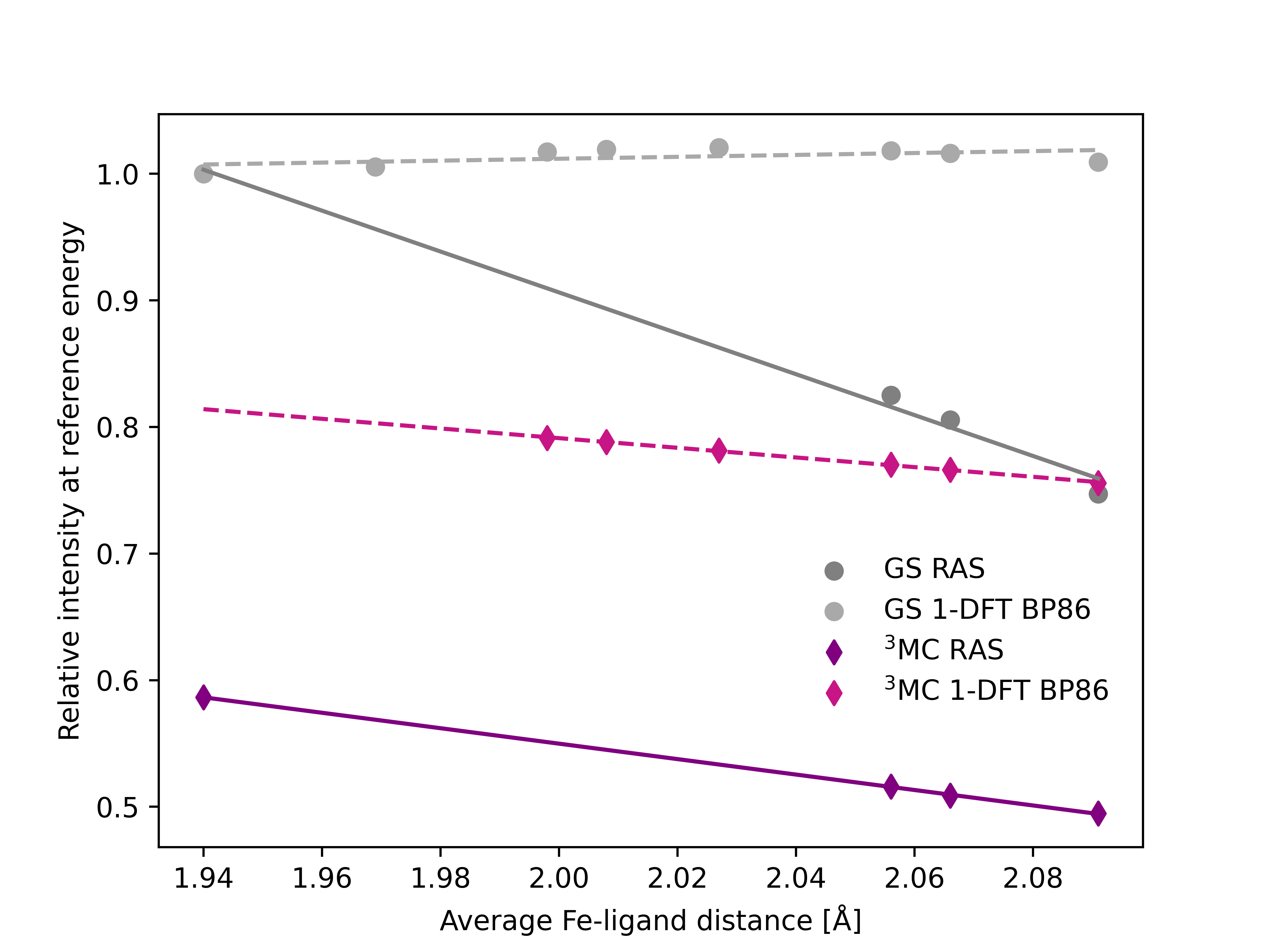

Supplement: CP-025-D2CP05671B-s001 [file CP-025-D2CP05671B-s001.zip › SI-figures/FigS16_dIdr_DFT_RAS_6_points_use_Kalpha.tif]

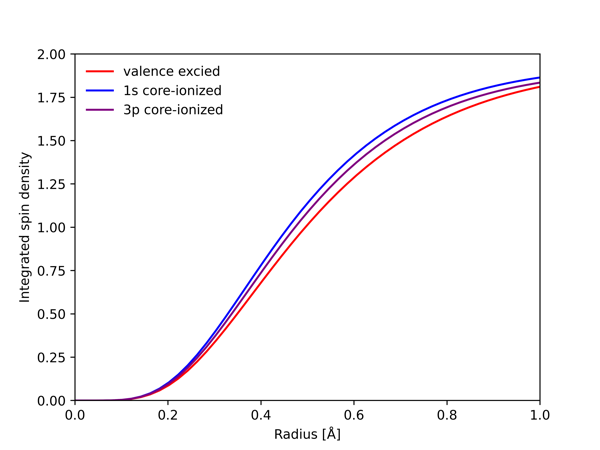

Supplement: CP-025-D2CP05671B-s001 [file CP-025-D2CP05671B-s001.zip › SI-figures/FigS17_spindensity_3d_noSig_short.png]

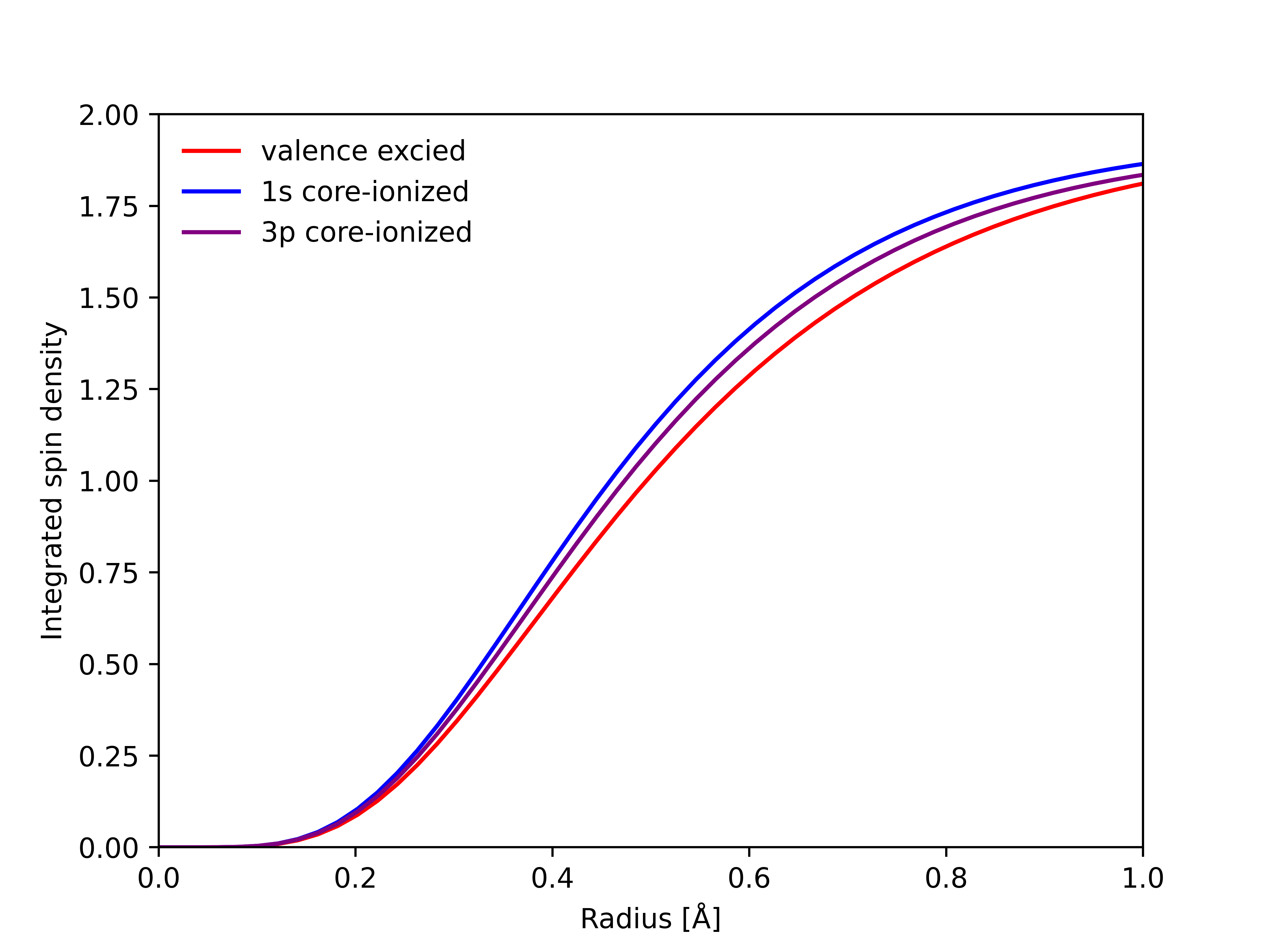

Supplement: CP-025-D2CP05671B-s001 [file CP-025-D2CP05671B-s001.zip › SI-figures/FigS17_spindensity_3d_noSig_short.tif]

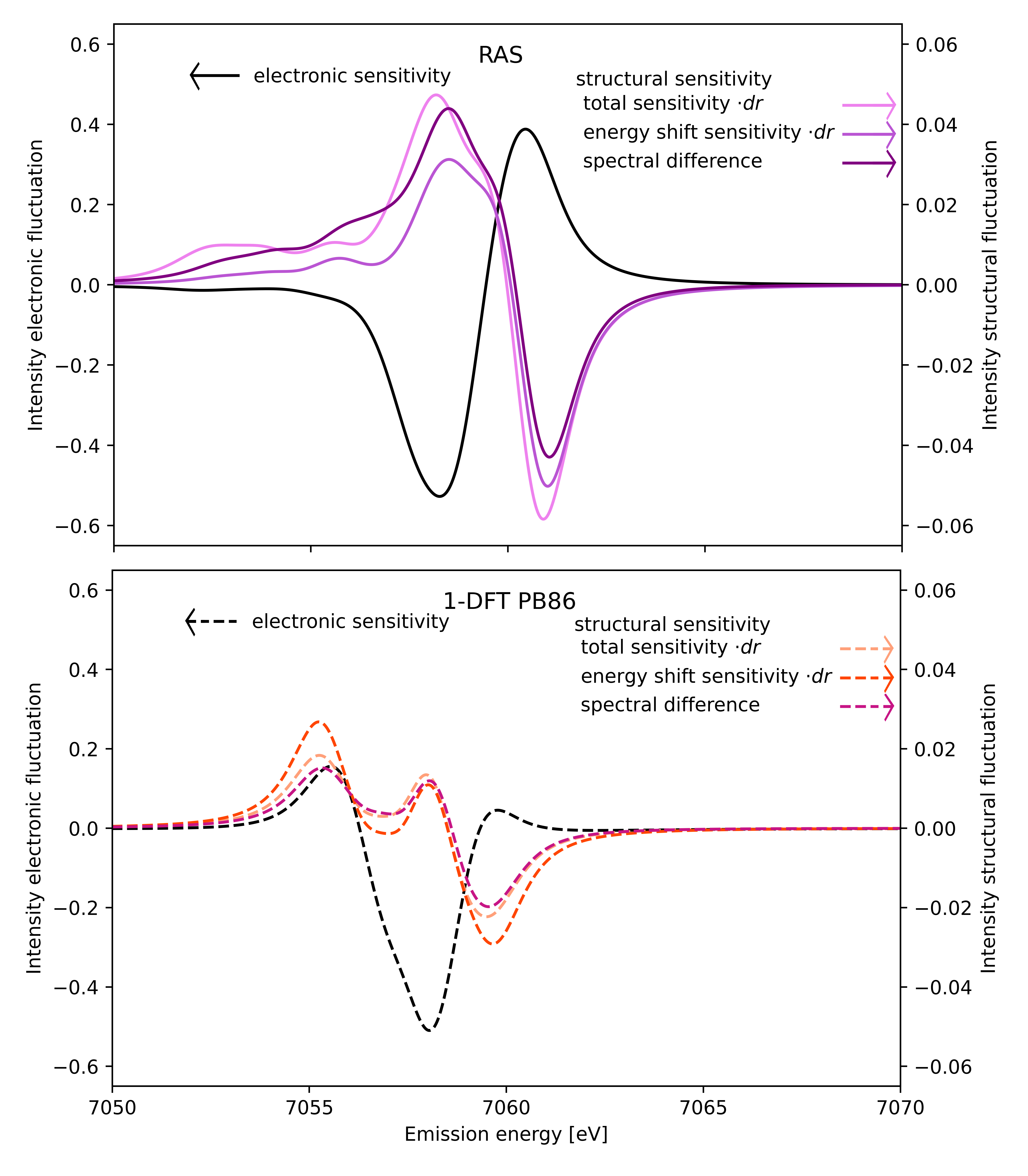

Supplement: CP-025-D2CP05671B-s001 [file CP-025-D2CP05671B-s001.zip › SI-figures/FigS18_struc_sens_RAS_DFT_arrow.png]

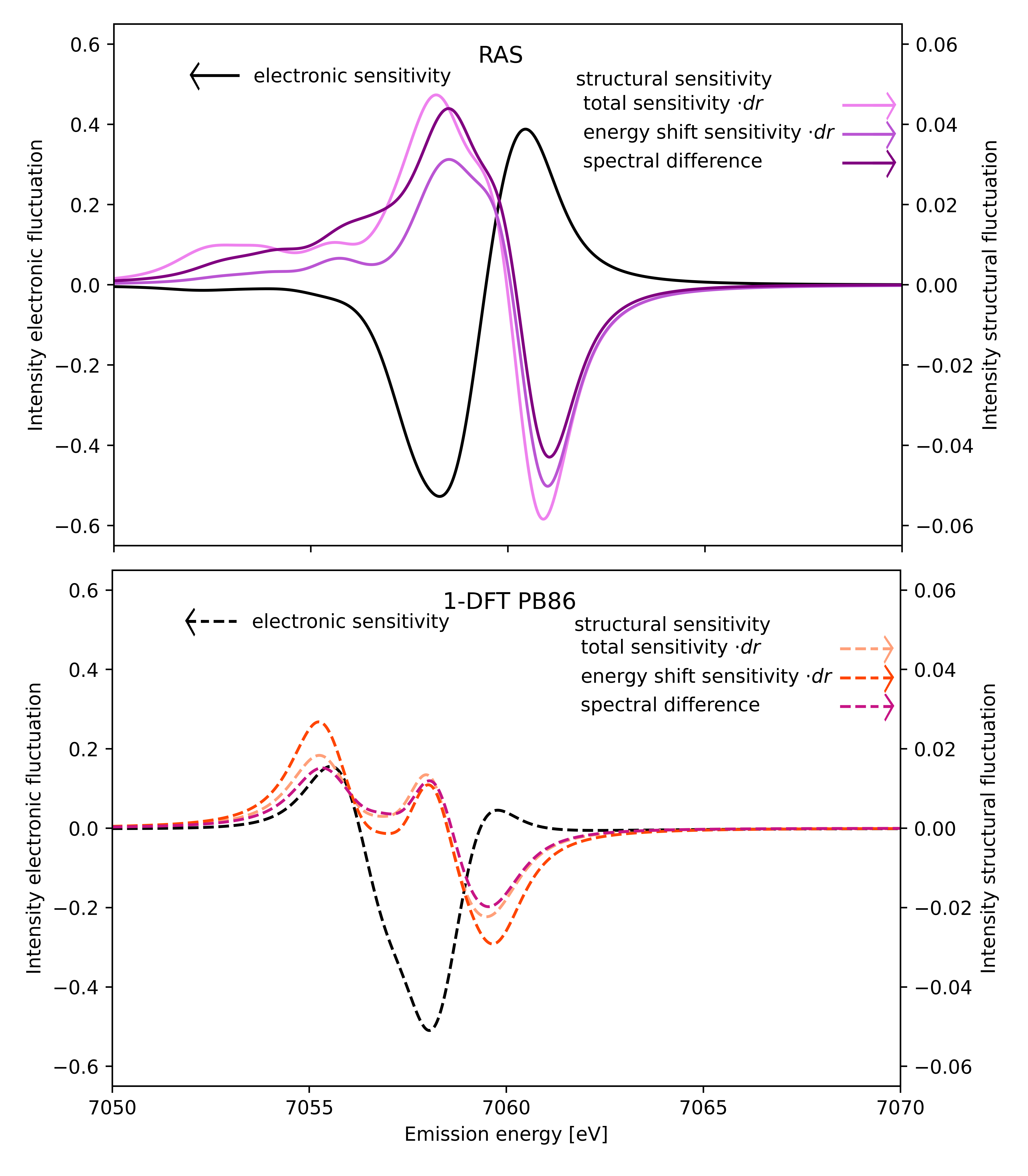

Supplement: CP-025-D2CP05671B-s001 [file CP-025-D2CP05671B-s001.zip › SI-figures/FigS18_struc_sens_RAS_DFT_arrow.tif]

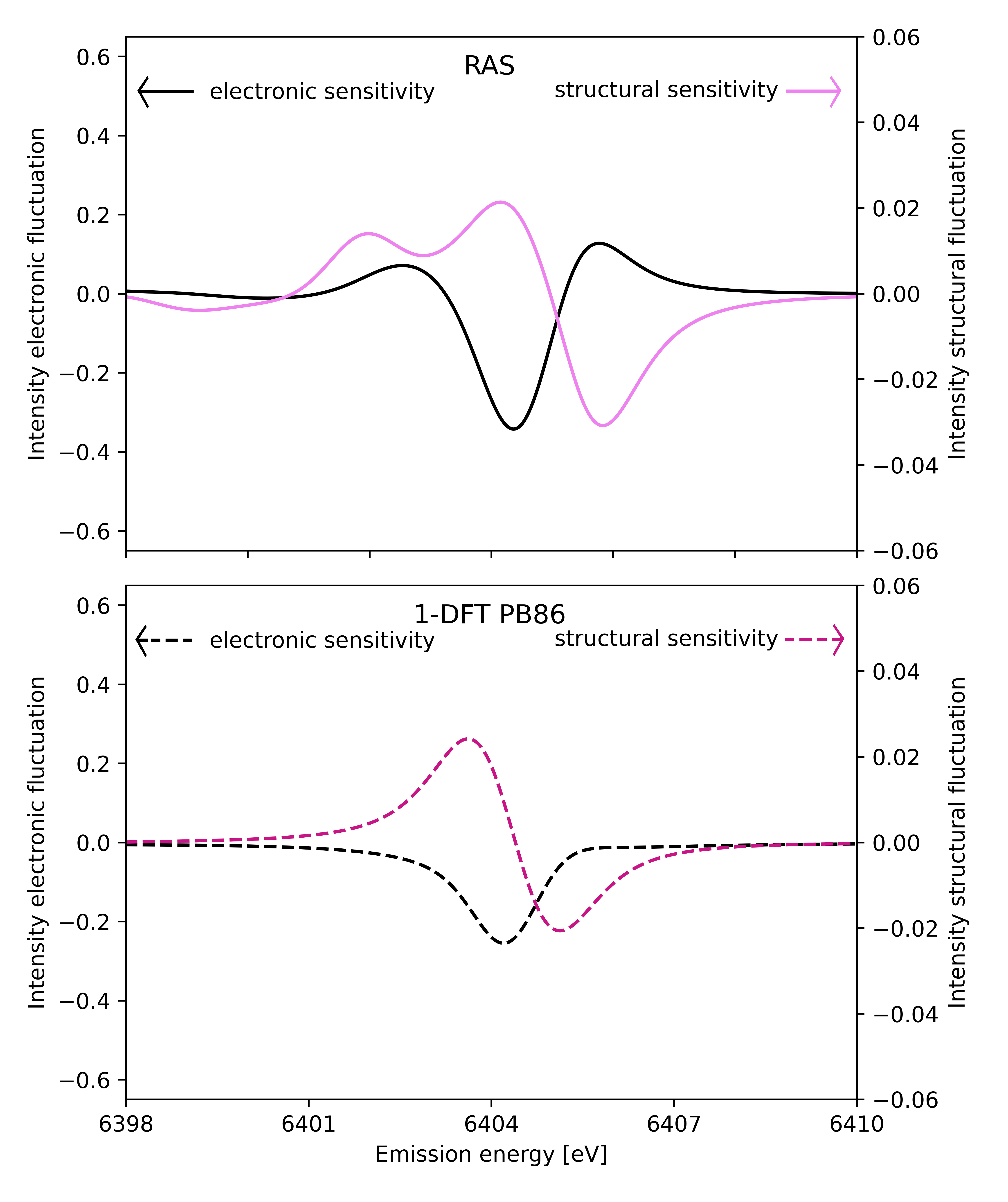

Supplement: CP-025-D2CP05671B-s001 [file CP-025-D2CP05671B-s001.zip › SI-figures/FigS19_struc_sens_Kalpha_RAS_DFT.png]

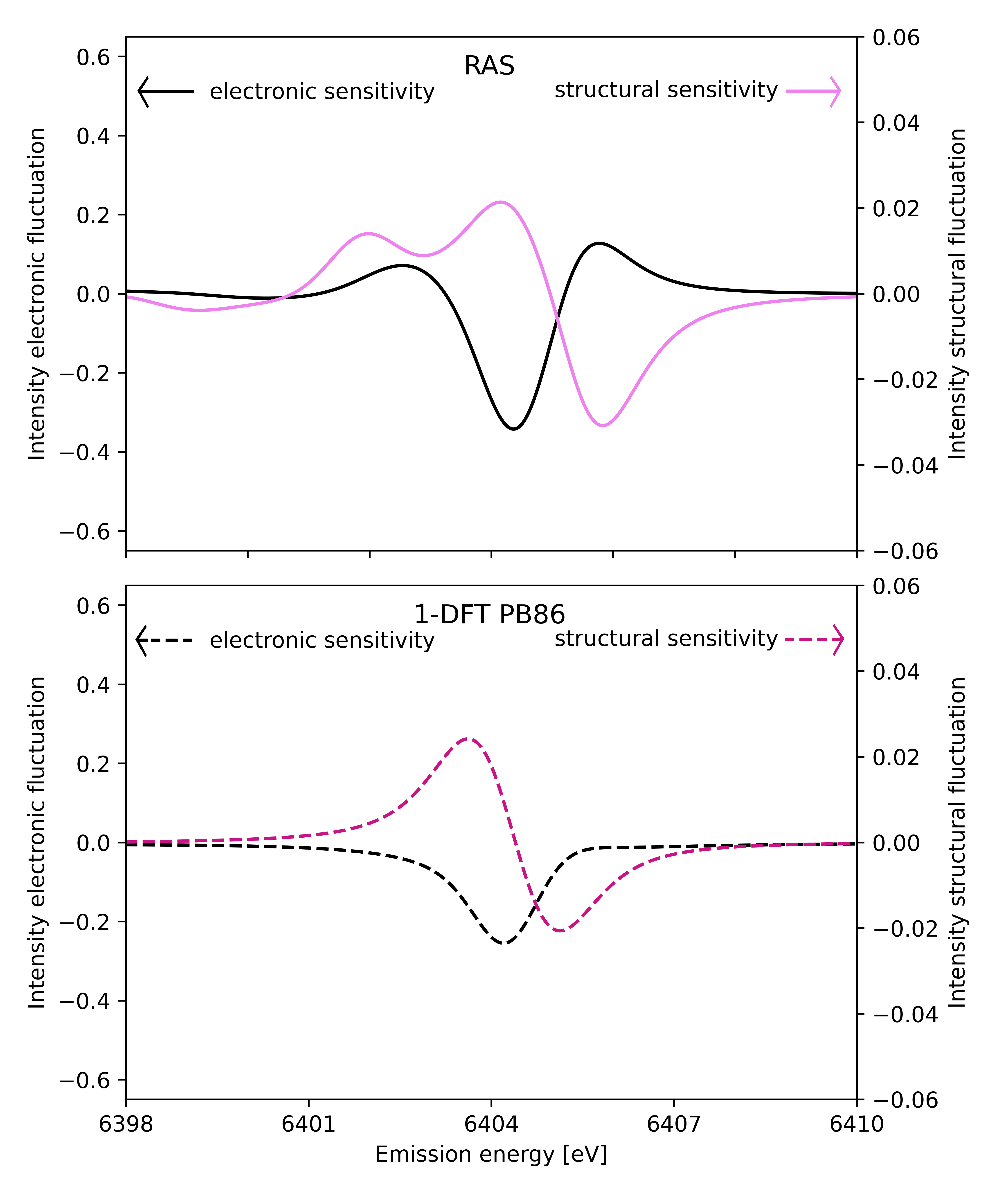

Supplement: CP-025-D2CP05671B-s001 [file CP-025-D2CP05671B-s001.zip › SI-figures/FigS19_struc_sens_Kalpha_RAS_DFT.tif]

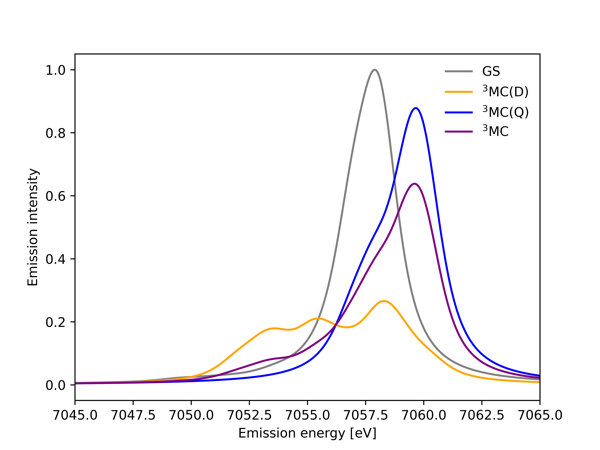

Supplement: CP-025-D2CP05671B-s001 [file CP-025-D2CP05671B-s001.zip › SI-figures/FigS2_GS-geom_all-states_spectra.png]

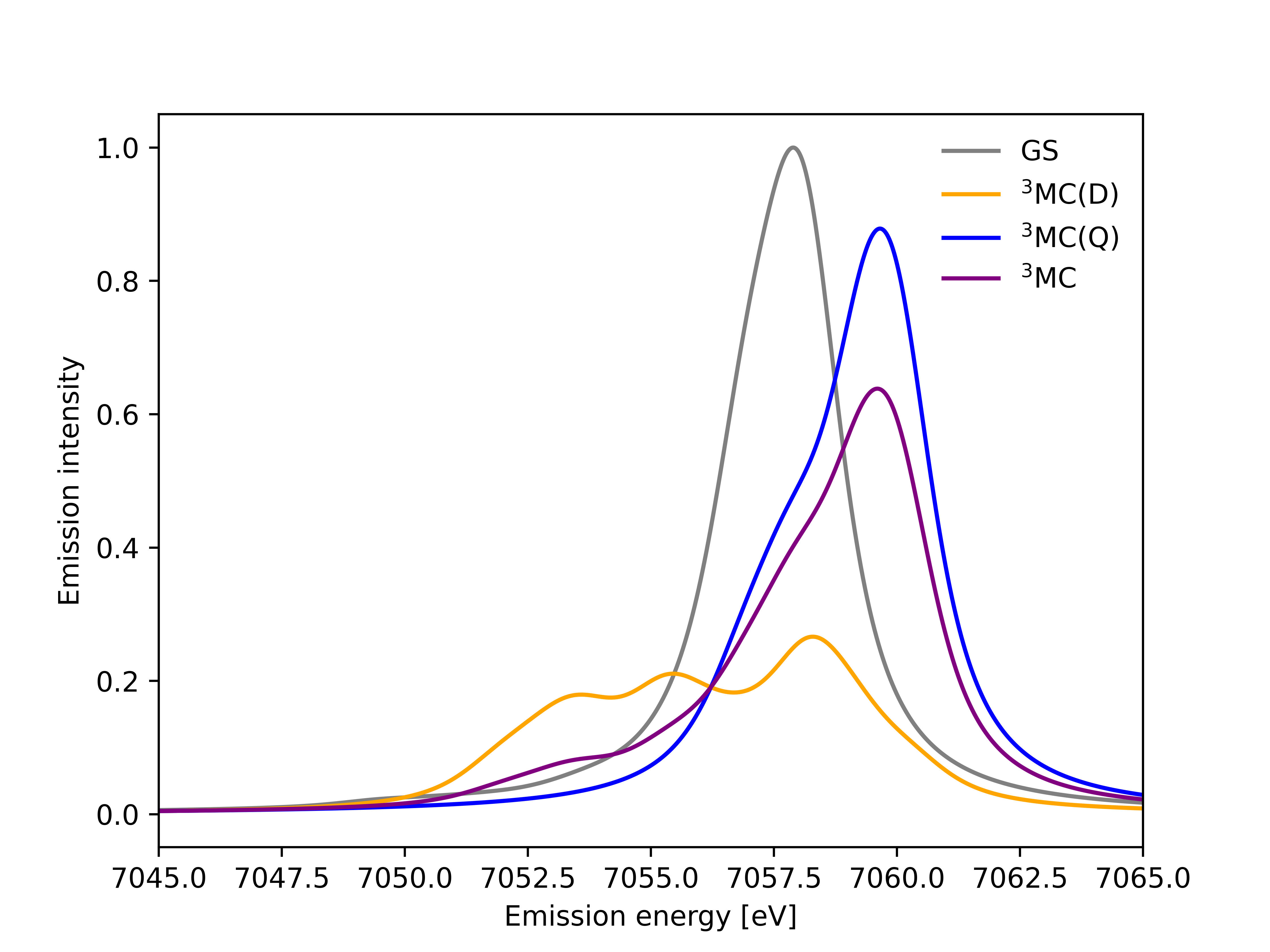

Supplement: CP-025-D2CP05671B-s001 [file CP-025-D2CP05671B-s001.zip › SI-figures/FigS2_GS-geom_all-states_spectra.tif]

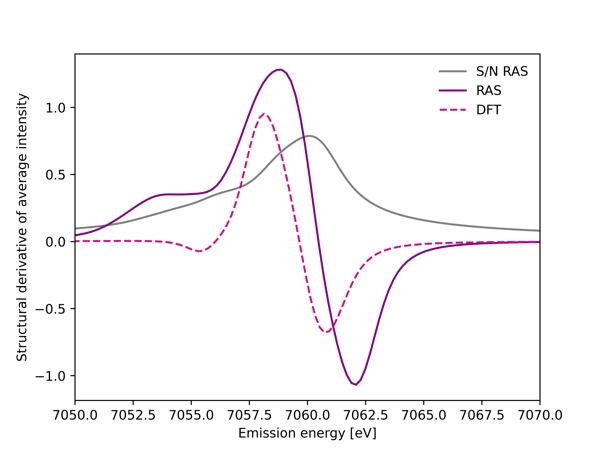

Supplement: CP-025-D2CP05671B-s001 [file CP-025-D2CP05671B-s001.zip › SI-figures/FigS20_struc_sens_average_SN_RAS_DFT_normalized.png]

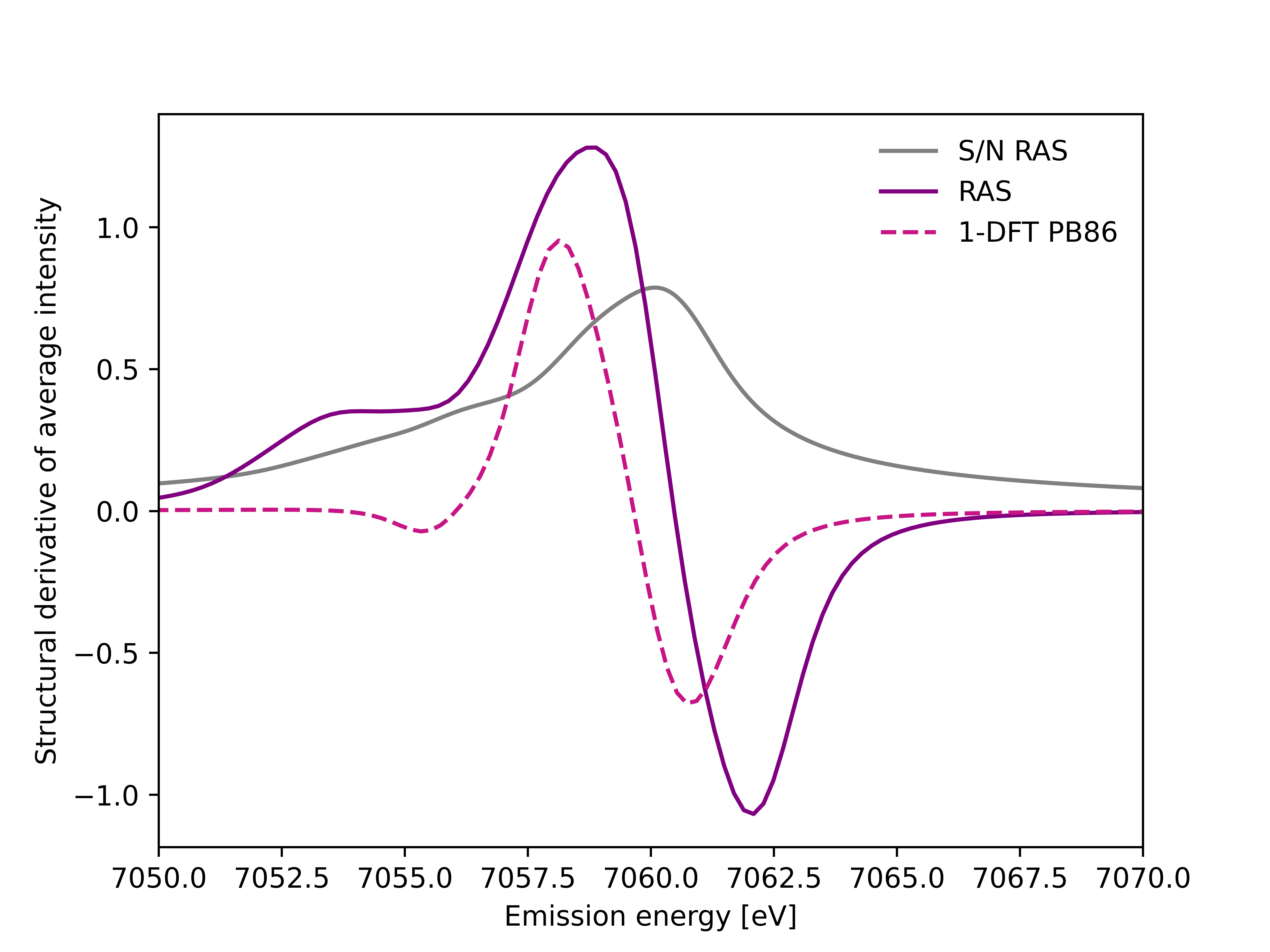

Supplement: CP-025-D2CP05671B-s001 [file CP-025-D2CP05671B-s001.zip › SI-figures/FigS20_struc_sens_average_SN_RAS_DFT_normalized.tif]

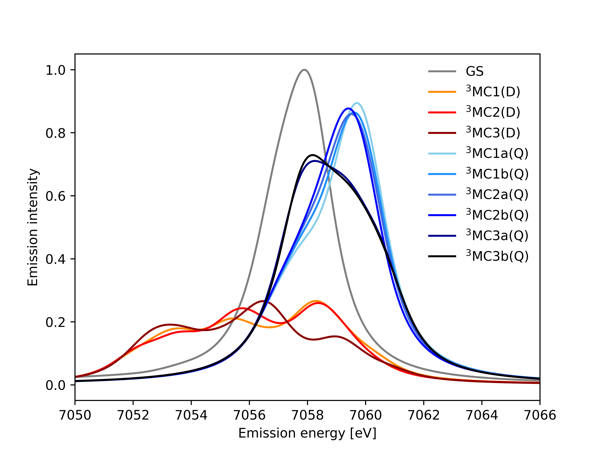

Supplement: CP-025-D2CP05671B-s001 [file CP-025-D2CP05671B-s001.zip › SI-figures/FigS3_GS-geom_all-states_grey.png]

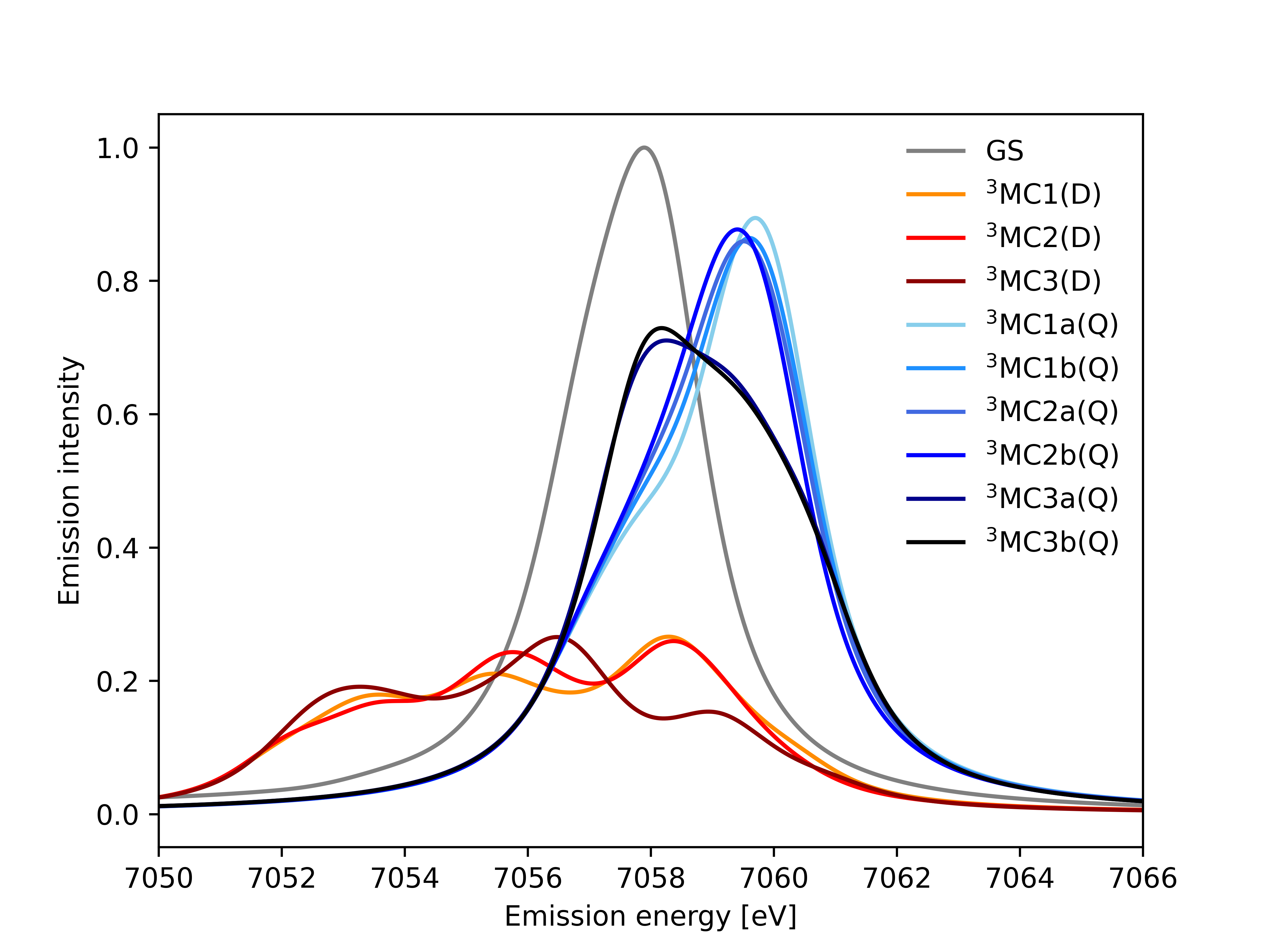

Supplement: CP-025-D2CP05671B-s001 [file CP-025-D2CP05671B-s001.zip › SI-figures/FigS3_GS-geom_all-states_grey.tif]

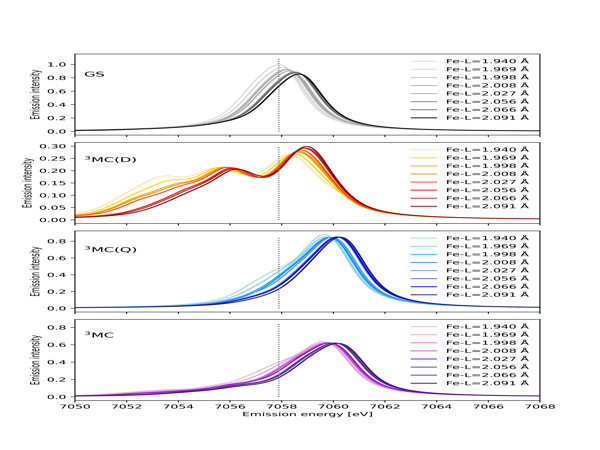

Supplement: CP-025-D2CP05671B-s001 [file CP-025-D2CP05671B-s001.zip › SI-figures/FigS4_GS_3MCDQ_RAS.png]

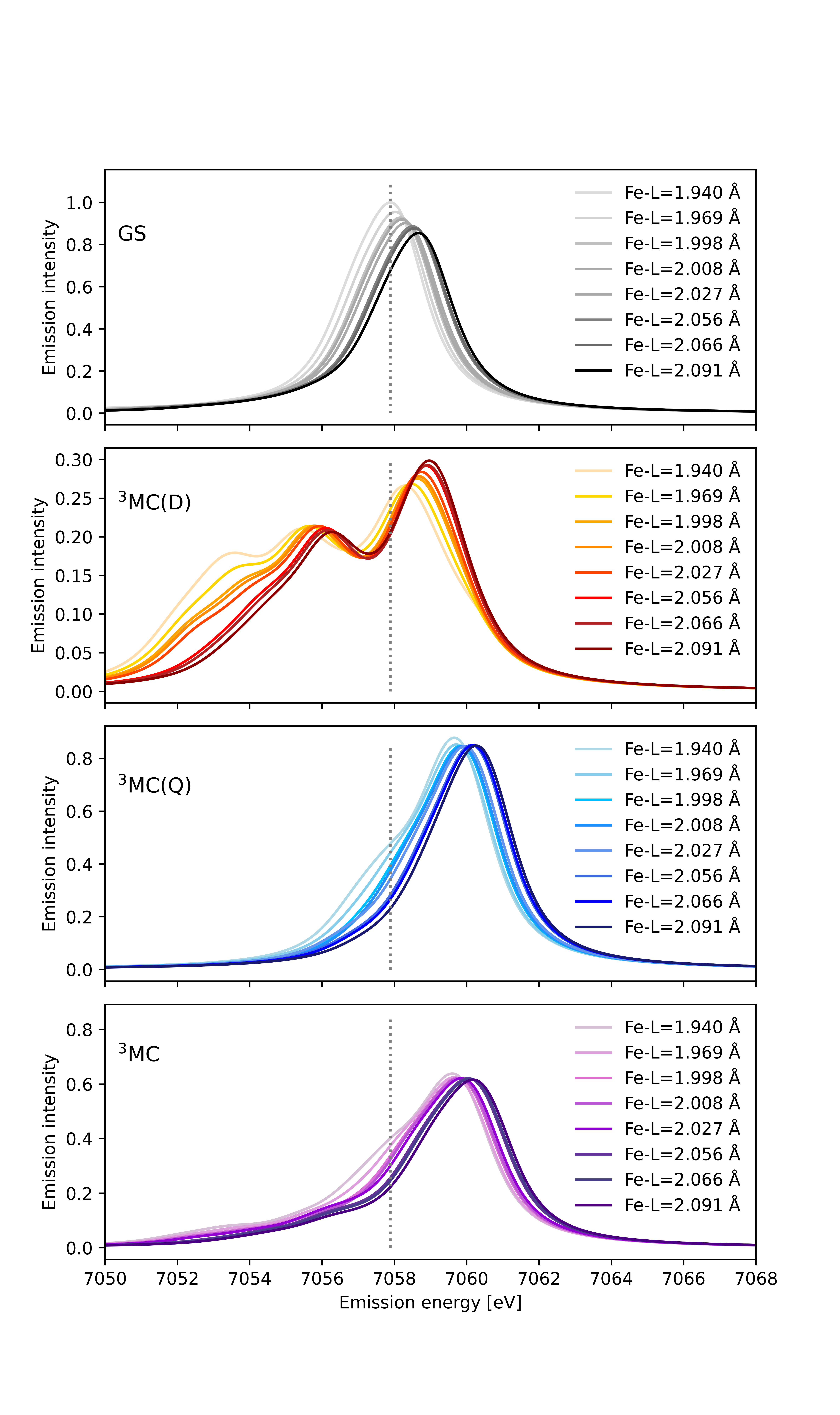

Supplement: CP-025-D2CP05671B-s001 [file CP-025-D2CP05671B-s001.zip › SI-figures/FigS4_GS_3MCDQ_RAS.tif]

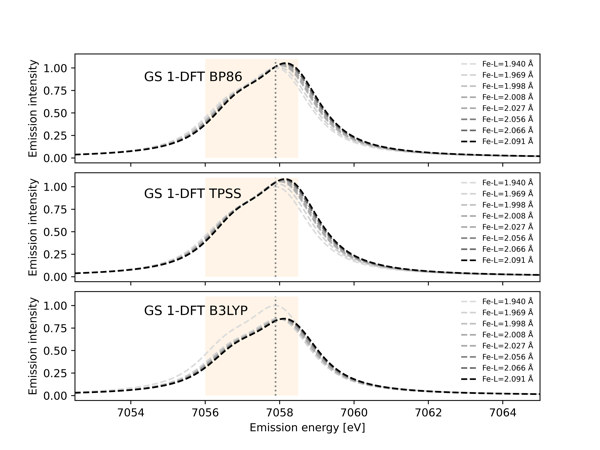

Supplement: CP-025-D2CP05671B-s001 [file CP-025-D2CP05671B-s001.zip › SI-figures/FigS5_GS_DFT_all_separate.png]

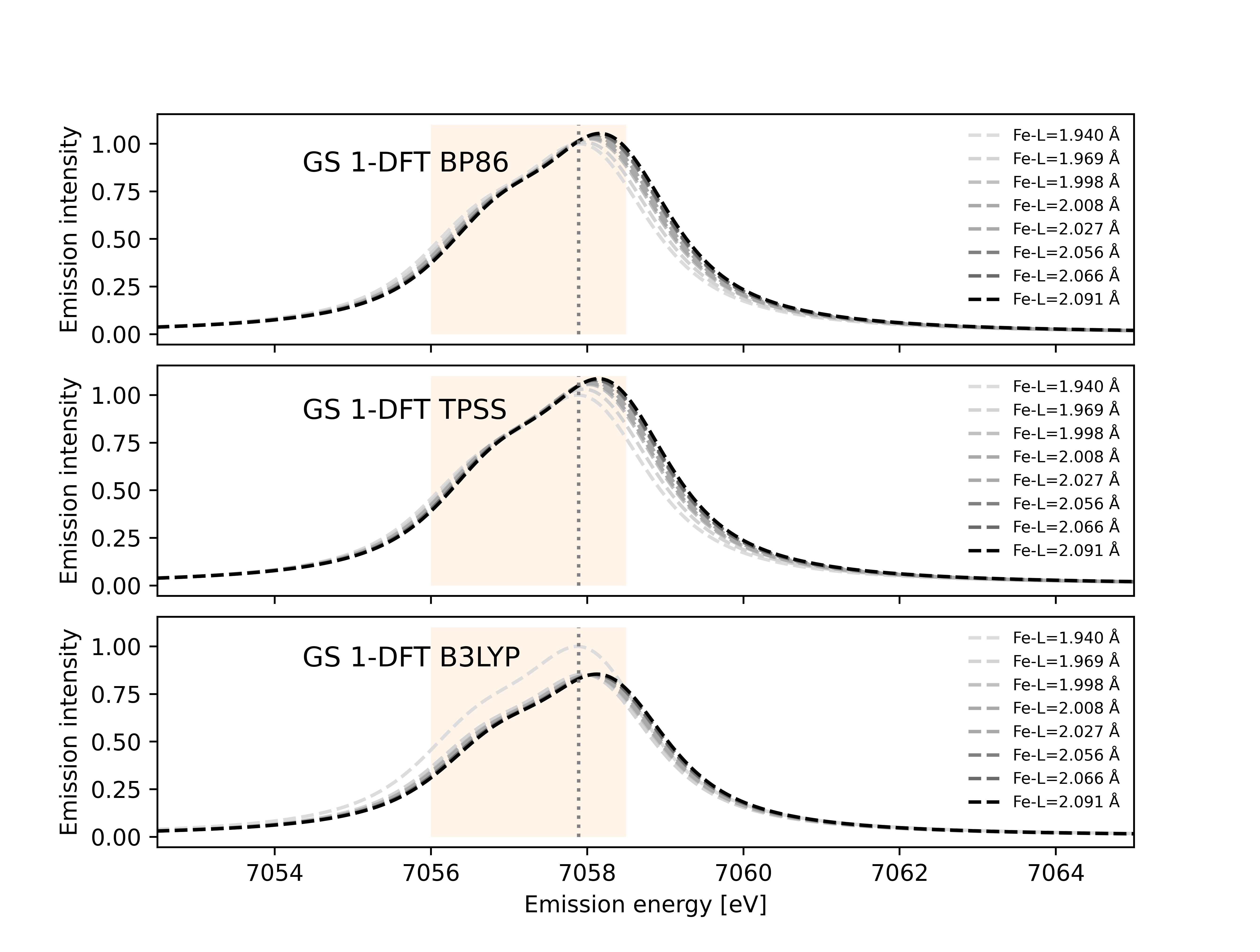

Supplement: CP-025-D2CP05671B-s001 [file CP-025-D2CP05671B-s001.zip › SI-figures/FigS5_GS_DFT_all_separate.tif]

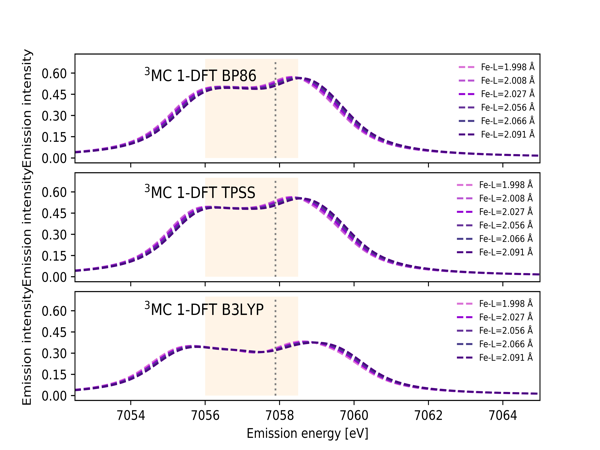

Supplement: CP-025-D2CP05671B-s001 [file CP-025-D2CP05671B-s001.zip › SI-figures/FigS6_3MC_DFT_all_separate_noGS_31.png]

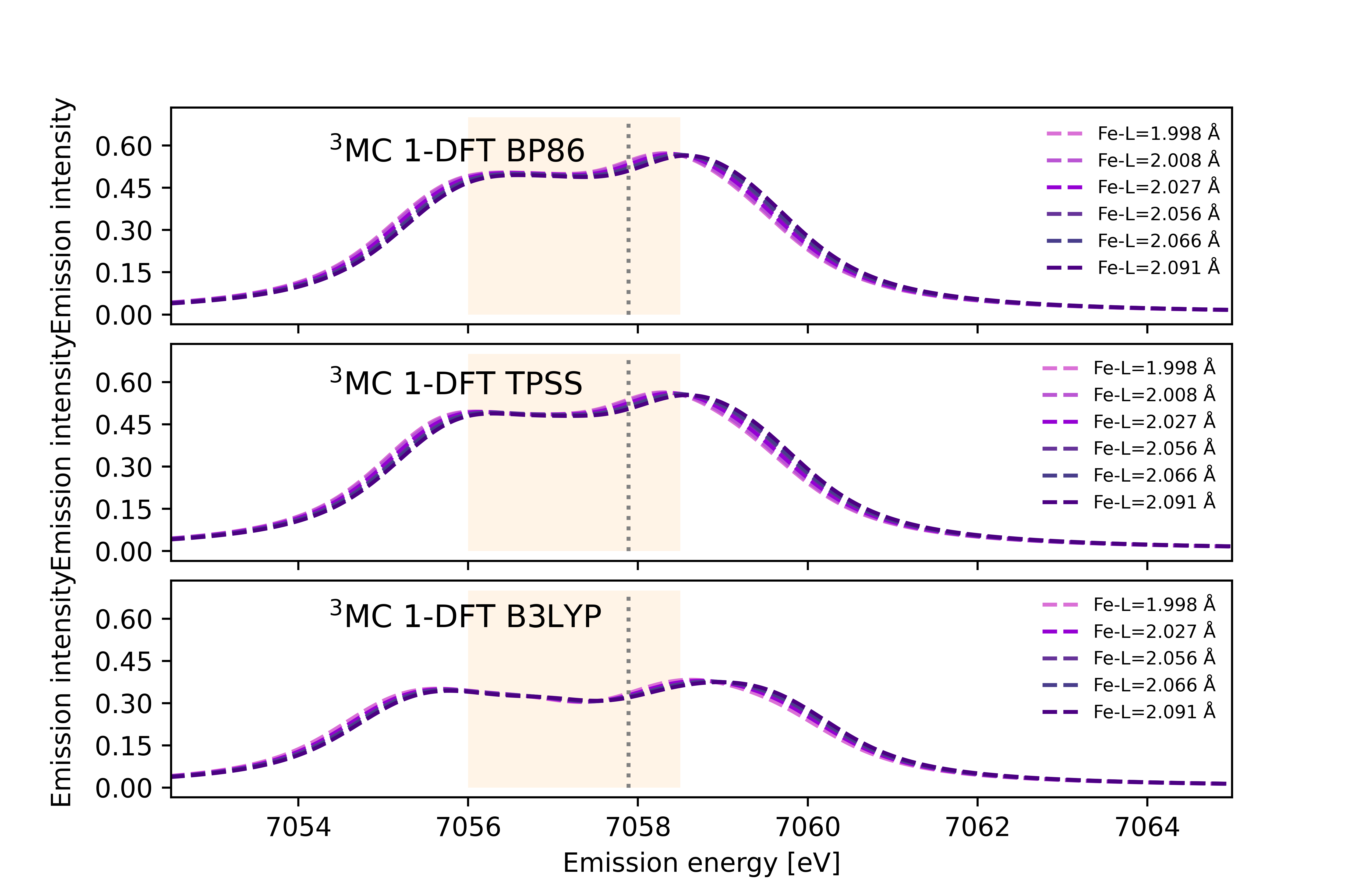

Supplement: CP-025-D2CP05671B-s001 [file CP-025-D2CP05671B-s001.zip › SI-figures/FigS6_3MC_DFT_all_separate_noGS_31.tif]

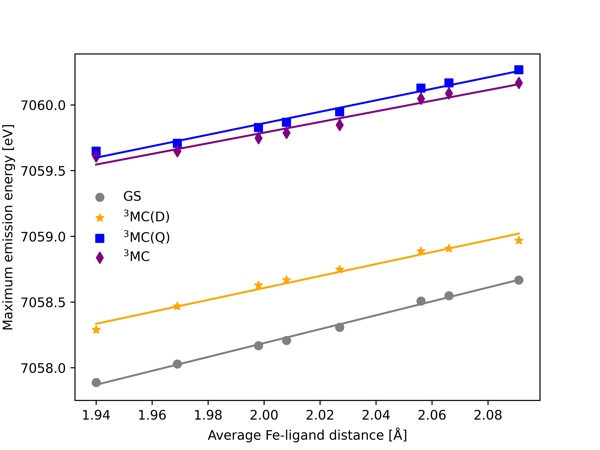

Supplement: CP-025-D2CP05671B-s001 [file CP-025-D2CP05671B-s001.zip › SI-figures/FigS7_dEdr_RAS_DQ.png]

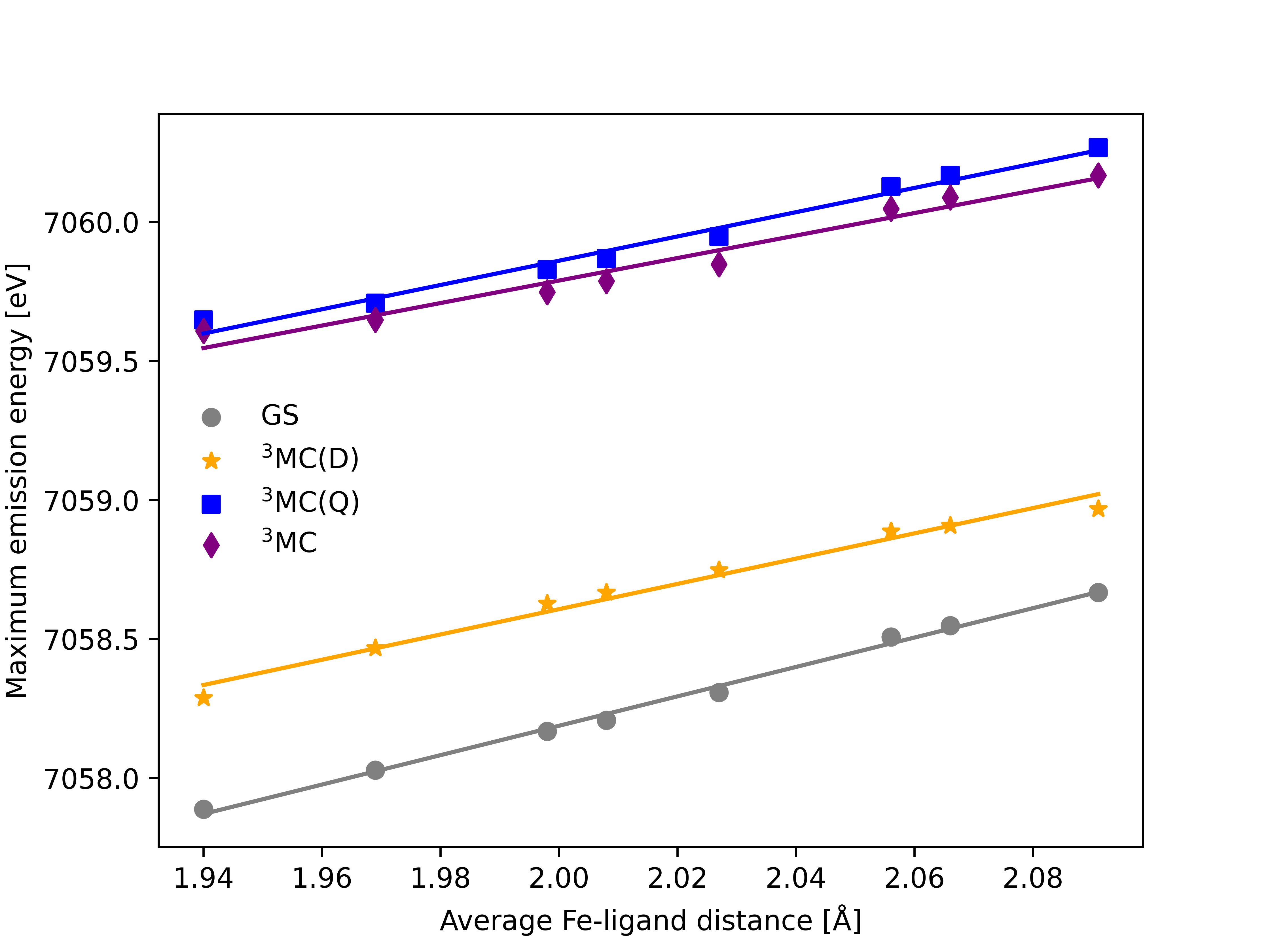

Supplement: CP-025-D2CP05671B-s001 [file CP-025-D2CP05671B-s001.zip › SI-figures/FigS7_dEdr_RAS_DQ.tif]

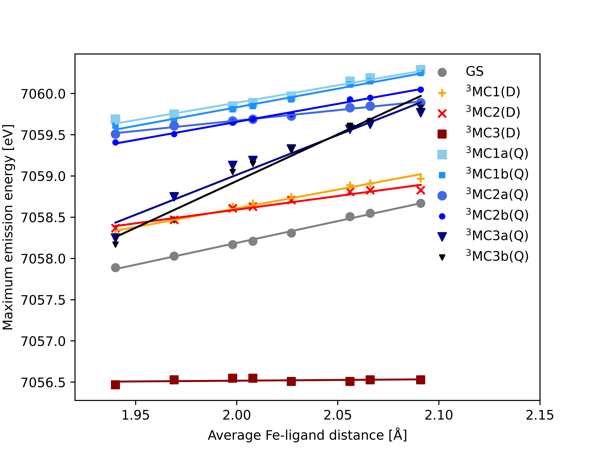

Supplement: CP-025-D2CP05671B-s001 [file CP-025-D2CP05671B-s001.zip › SI-figures/FigS8_dEdr_RAS_DQ_all.png]

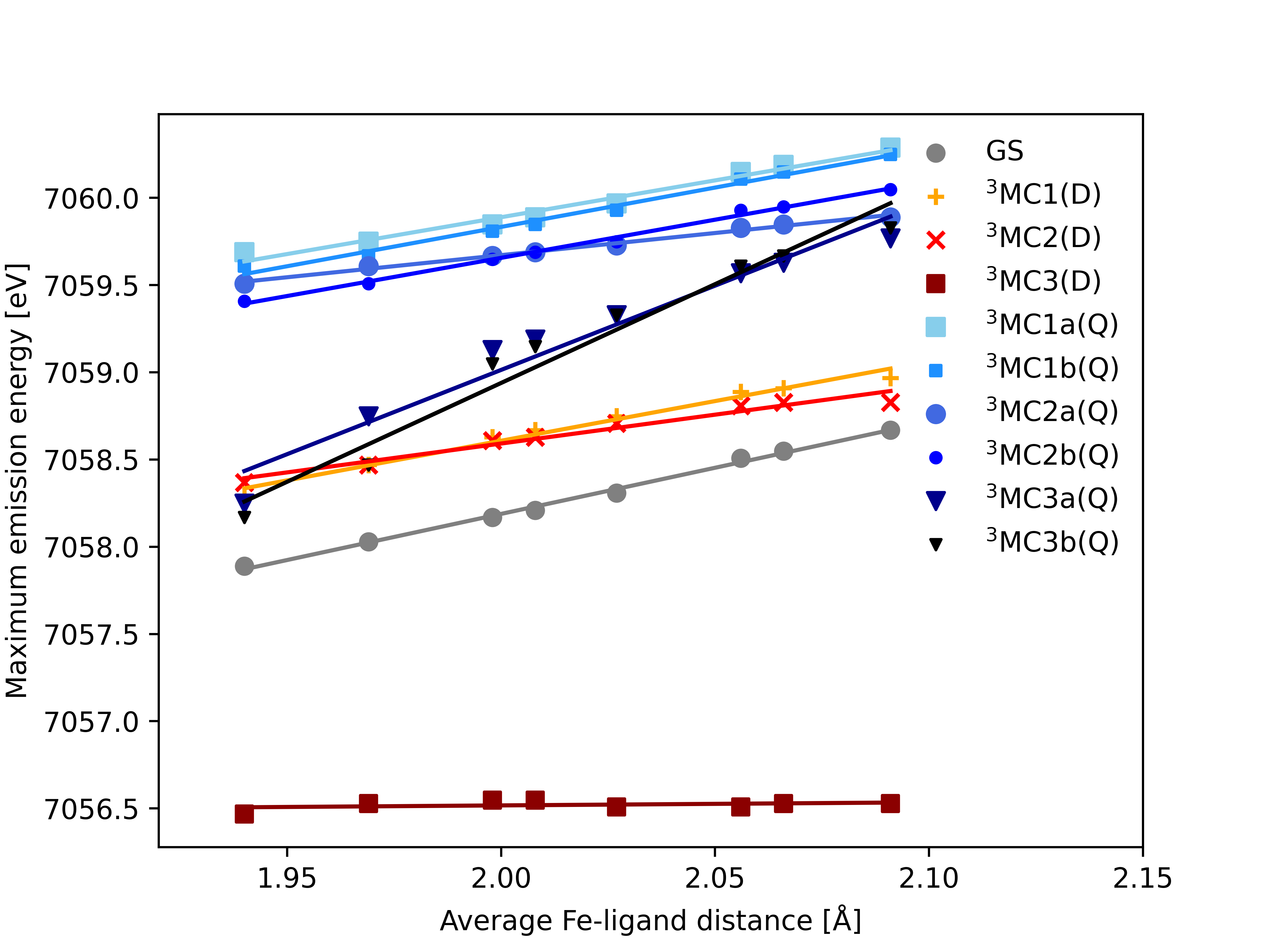

Supplement: CP-025-D2CP05671B-s001 [file CP-025-D2CP05671B-s001.zip › SI-figures/FigS8_dEdr_RAS_DQ_all.tif]

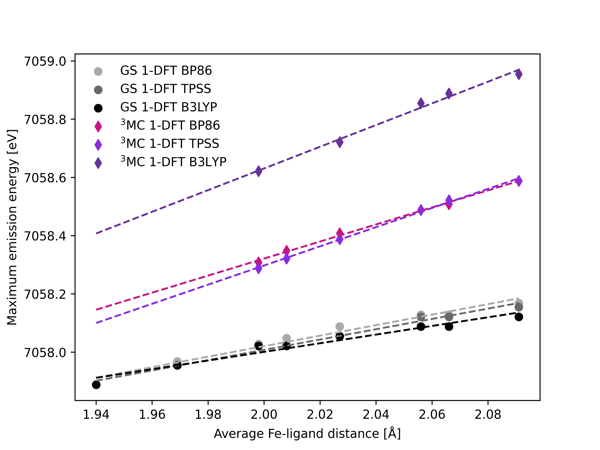

Supplement: CP-025-D2CP05671B-s001 [file CP-025-D2CP05671B-s001.zip › SI-figures/FigS9_dEdr_DFT_All.png]

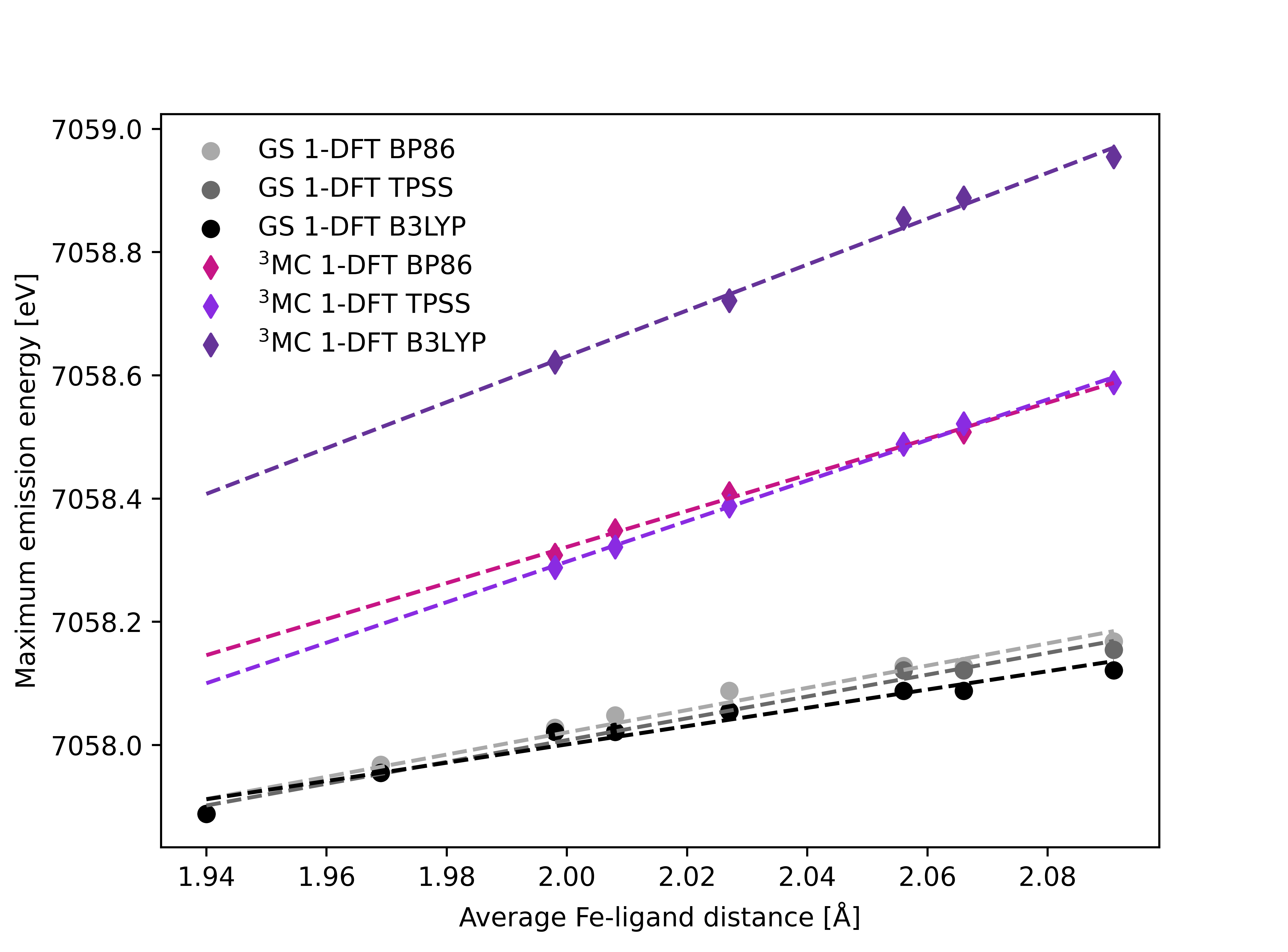

Supplement: CP-025-D2CP05671B-s001 [file CP-025-D2CP05671B-s001.zip › SI-figures/FigS9_dEdr_DFT_All.tif]
