# Supplementary material for: Sensitivity of Kβ mainline X-ray emission to structural dynamics in iron photosensitizer
Source: Phys Chem Chem Phys. 2023 Mar 30;25(15):10447–59. doi: 10.1039/d2cp05671b (PMC10091363; doi:10.1039/d2cp05671b)
Supplement: CP-025-D2CP05671B-s002 [file CP-025-D2CP05671B-s002.pdf]

This journal is © the Owner Societies 2023

Supplementary information: Sensitivity of Kβ mainline X-ray emission to structural dynamics in iron photosensitizer

Johanna Rogvall, Roshan Singh, Morgane Vacher, Marcus Lundberg

Contents

1 Additional figures **1**

2 Additional tables **9**

1 Additional figures

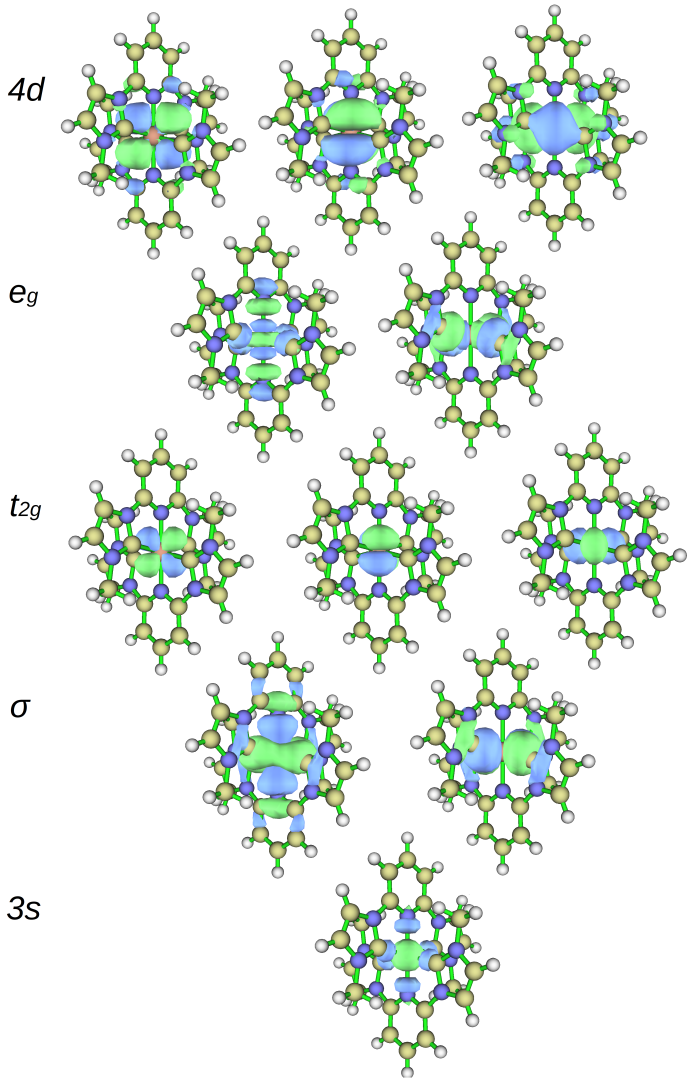

Figure S1: Isodensity plots of the RAS2 active orbitals: $3s$, σ, t_{2g}, e_g and $4d$. The orbitals are from a 1s core-hole calculation at the ground state geometry with doublet spin multiplicity.

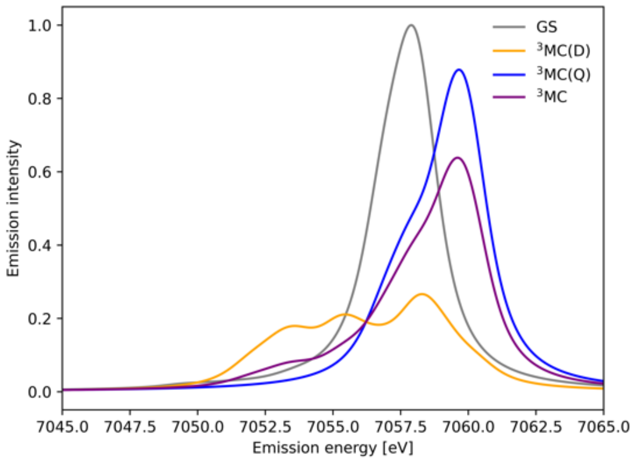

Figure S2: RAS Kβ emission spectra for GS and ^{3}MC states. The ^{3}MC spectrum is obtained from the emission from doublet (^{3}MC(D)) and quartet (^{3}MC(Q)) 1s core-ionized states with a 4:2 ratio. All spectra are simulated using the GS equilibrium geometry.

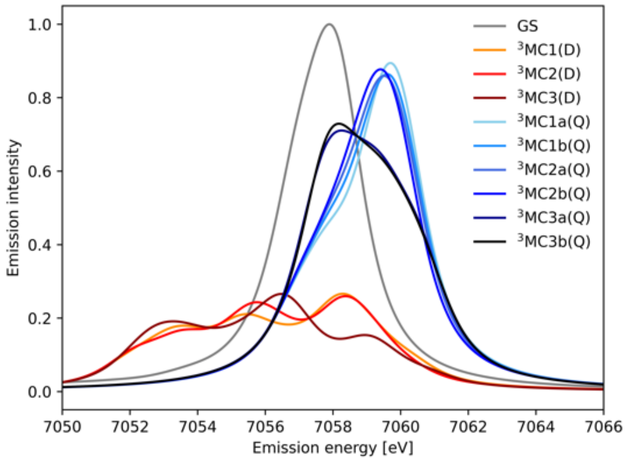

Figure S3: RAS Kβ emission spectra for low-energy ^{3}MC intermediate (1s core ionized) states calculated for the GS geometry. Quartet states are slit by spin-orbit coupling into groups of close-lying states (a and b). ^{3}MCX(Q) results are obtained using a 1:1 ratio of ^{3}MCXa(Q) and ^{3}MCXb(Q) results.

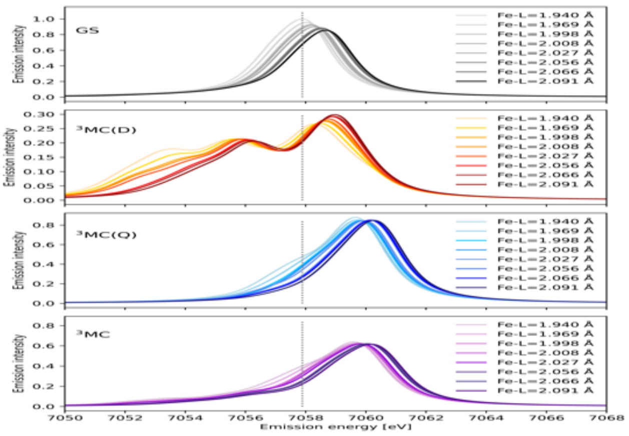

Figure S4: Structural sensitivity of RAS Kβ emission spectra from different core-ionized intermediate states. Data are shown for for GS (top), ^{3}MC(D) (second top), ^{3}MC(Q) (second bottom) and the combined ^{3}MC (bottom) at eight different geometries with increasing Fe-ligand distance. The vertical dashed line represent the maximum emission intensity of the experimental spectra 7057.9 eV. Note the different emission intensity scales of the individual plots.

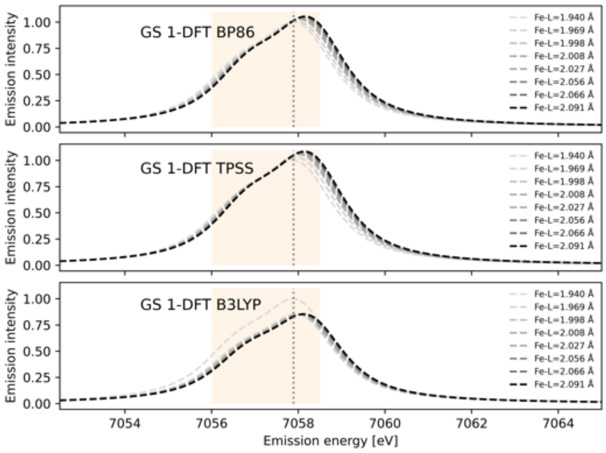

Figure S5: Functional dependence of simulated Kβ emission spectra for the GS electronic state with 1-DFT BP86, B3LYP and TPSS. The grey dotted vertical line at 7057.9 eV represents the maximum emission intensity of the experimental GS spectrum. The yellow area represents the 7056 eV – 7058.5 eV energy range used for the Kβ difference spectrum in reference[1].

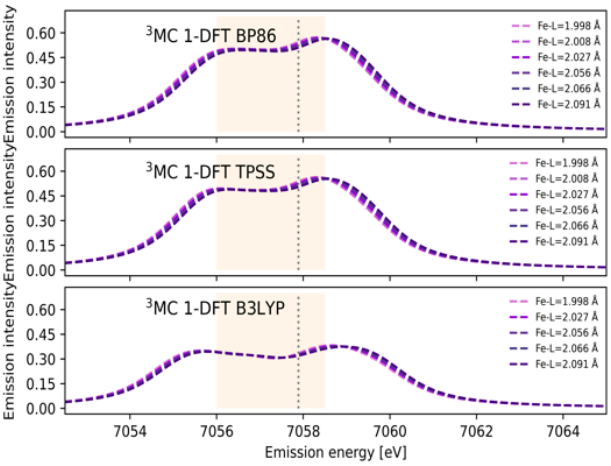

Figure S6: Functional dependence of simulated Kβ emission spectra for the electronic ^{3}MC state with 1-DFT BP86, B3LYP and TPSS. At short Fe-ligand distances the ^{3}MC state is not stable with respect to a ^{3}MLCT state and the corresponding spectra are not included. The grey dotted vertical line at 7057.9 eV represents the maximum emission intensity of the experimental GS spectrum. The yellow area represents the 7056 eV – 7058.5 eV energy range used for the Kβ difference spectrum in reference[1].

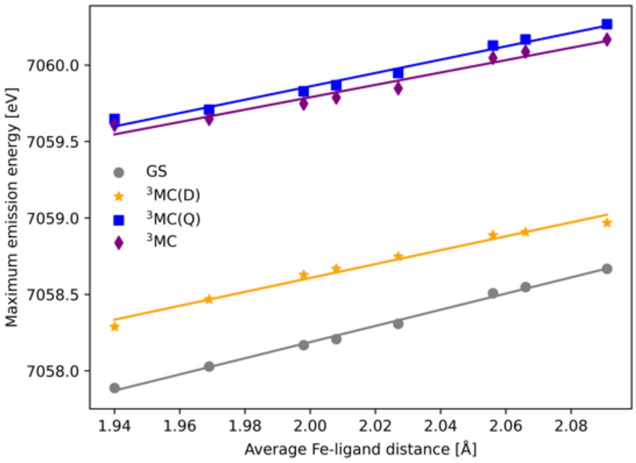

Figure S7: Energies of the RAS Kβ emission maxima as a function of the Fe-ligand distance for the GS, ^{3}MC(Q),^{3}MC(D) and the combined ^{3}MC states.

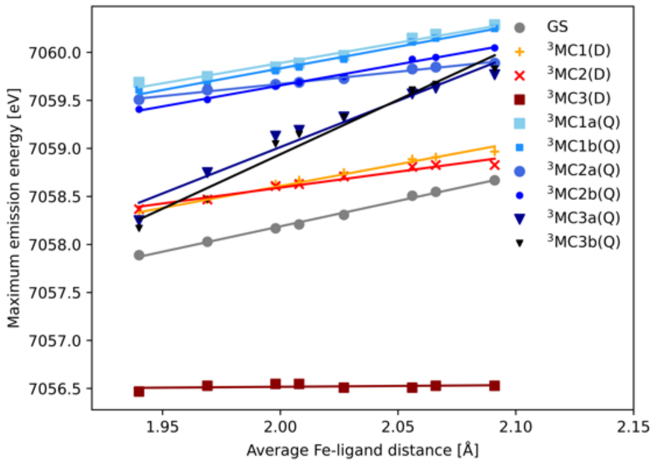

Figure S8: Energies of the RAS Kβ emission maxima as a function of the Fe-ligand distance for the three lowest ^{3}MC(Q) and ^{3}MC(D) energy states. ^{3}MCX(Q) results are obtained using a 1:1 ratio of ^{3}MCXa(Q) and ^{3}MCXb(Q) results.

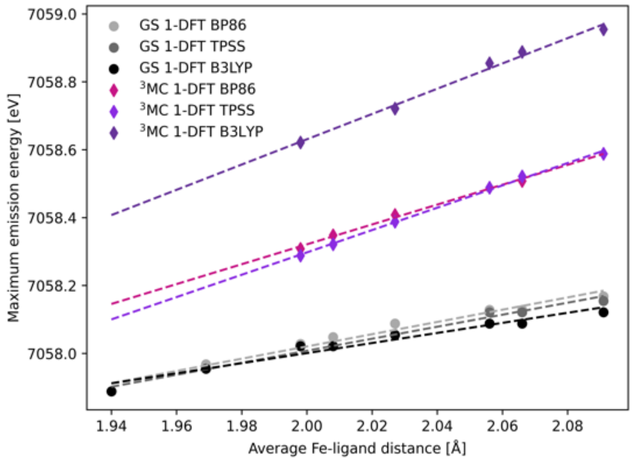

Figure S9: Functional dependence of the energies of the 1-DFT Kβ emission maxima of the ^{3}MC and GS electronic states as a function of the Fe-ligand distance. Linear regression lines were fitted for each set of calculations.

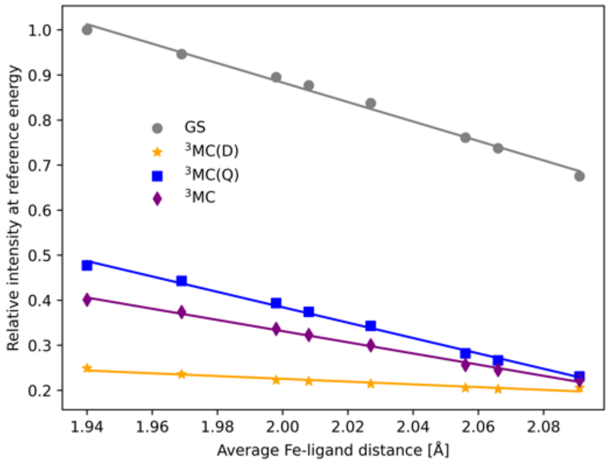

Figure S10: RAS Kβ emission intensities at the GS maximum energy 7057.9 eV, as a function of the Fe-ligand distance for the GS, ^{3}MC(Q),^{3}MC(D) and the combined ^{3}MC states.

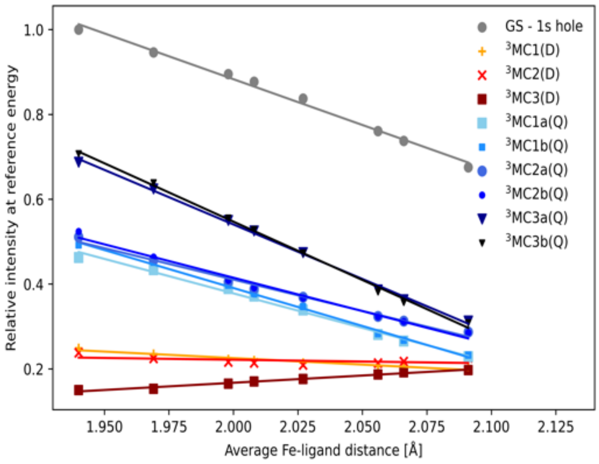

Figure S11: Integrated RAS Kβ emission intensities at the GS maximum energy 7057.9 eV, as a function of the Fe-ligand distance for the GS, ^{3}MC(D) and ^{3}MC(Q) states. Quartet states are slit by spin-orbit coupling into groups of close-lying states (a and b). The ^{3}MCXa(Q) and ^{3}MCXb(Q) states are combined 1:1 to form the ^{3}MCX(Q) state.

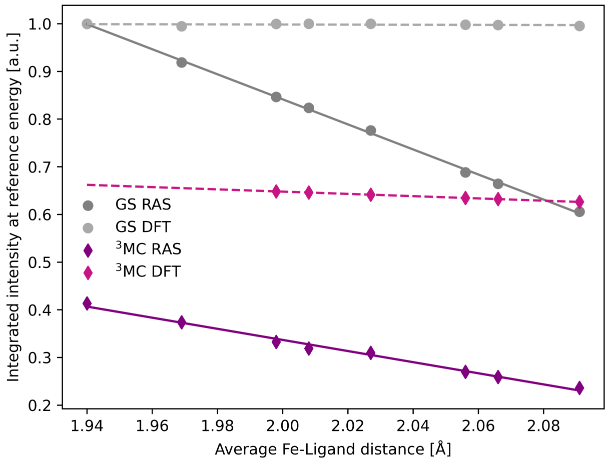

Figure S12: Kβ emission intensities for the 7056-7058.5 eV energy range used in reference [1], as a function of the Fe-ligand distance for the GS and ^{3}MC states for both RAS and 1-DFT BP86.

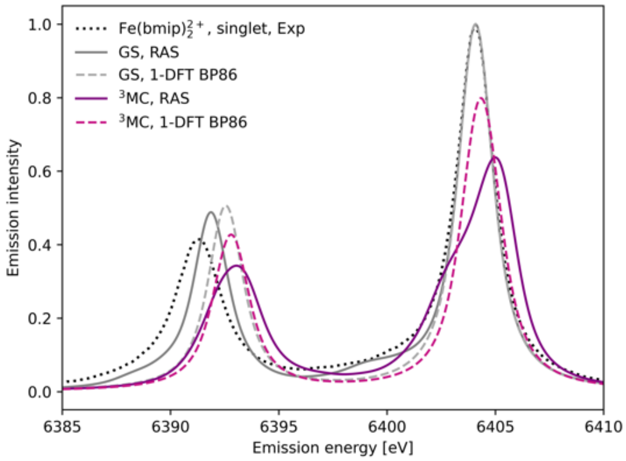

Figure S13: Kα x-ray emission spectra from experiment, RAS and 1-DFT BP86 simulations. The GS and ^{3}MC spectra are calculated at the GS and ^{3}MC equilibrium geometies respectively. RAS data are taken from reference [2].

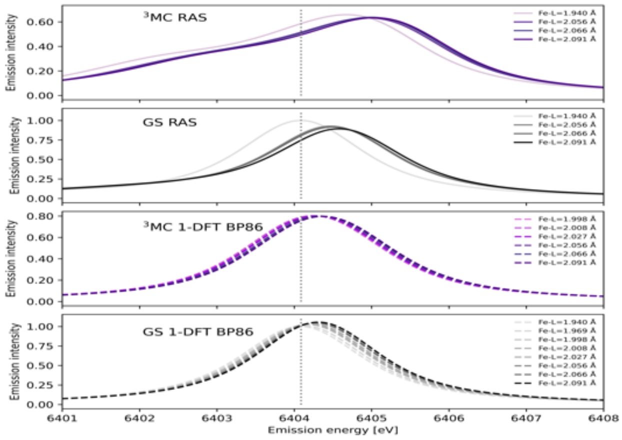

Figure S14: Kα emission spectra simulated using RAS and 1-DFT BP86 for GS (top) and ^{3}MC (bottom) electronic structures at different geometries with increasing Fe-ligand distances. The vertical dashed line represent the maximum emission intensity of the experimental spectra 6404.3 eV. RAS data are taken from reference[2].

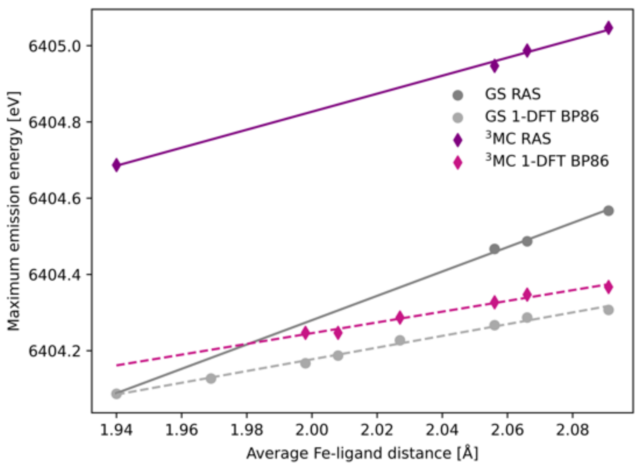

Figure S15: Energies of the Kα emission maxima as a function of the Fe-ligand distance for the GS and ^{3}MC from RAS (reference[2]) and 1-DFT BP86 simulations.

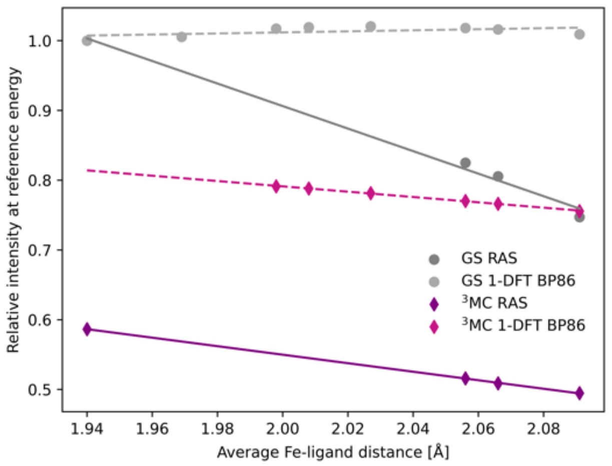

Figure S16: Intensities at the Kα GS emission maxima as a function of the Fe-ligand distance for the GS and ^{3}MC from RAS (referenence[2]) and 1-DFT BP86 simulations.

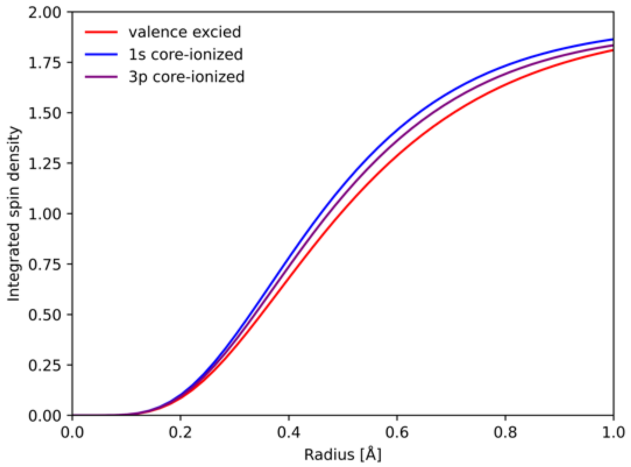

Figure S17: Integrated RAS radial spin density (RSD) plots of the ^{3}MC valence state, and the corresponding states with $1s$ and $3p$ holes calculated at the ^{3}MC equilibrium geometry. To avoid effects of changes in core orbital occupation, only the Fe $3d$ t_{2g} and e_g orbitals were included.

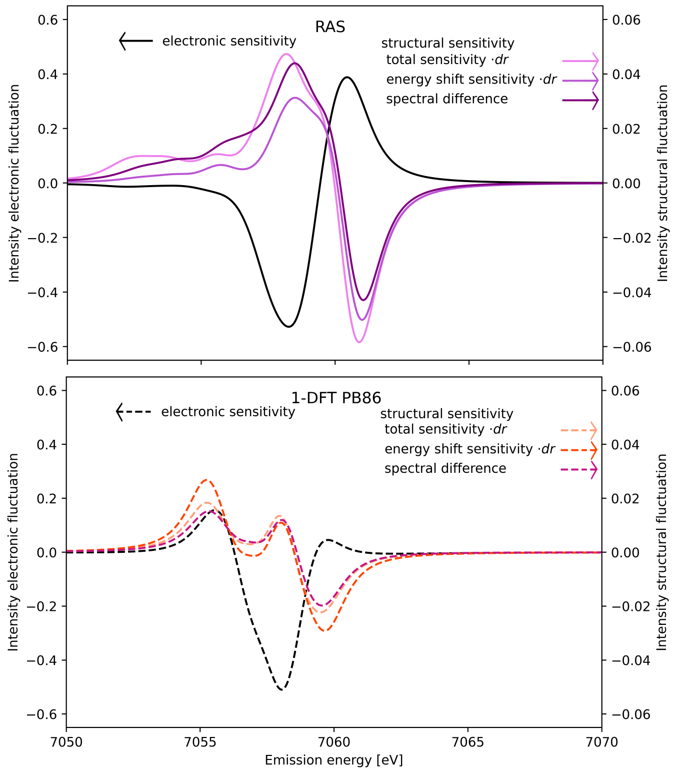

Figure S18: Comparison of three ways to estimate Kβ structural sensitivity of the ^{3}MC electronic state as a function of the emission energy. i) Slope of the intensity as a function of Fe-ligand distance (Total intensity) multiplied with the difference in the Fe-L bond length at the turning points in the wavepacket simulations (Δr=0.035 Å) ($dI/dr \cdot \Delta r$). ii) Energy shift sensitivity ($\delta E_{max}/\delta r \cdot dI/dE$) at the ^3MC minimum geometry multiplied with vibrational oscillation (Δr). This is the same method used in reference[2]. iii) Intensity difference between the the turning points in wavepacket simulations (Fe-L distances of 2.056 Å and 2.091 Å respectively).

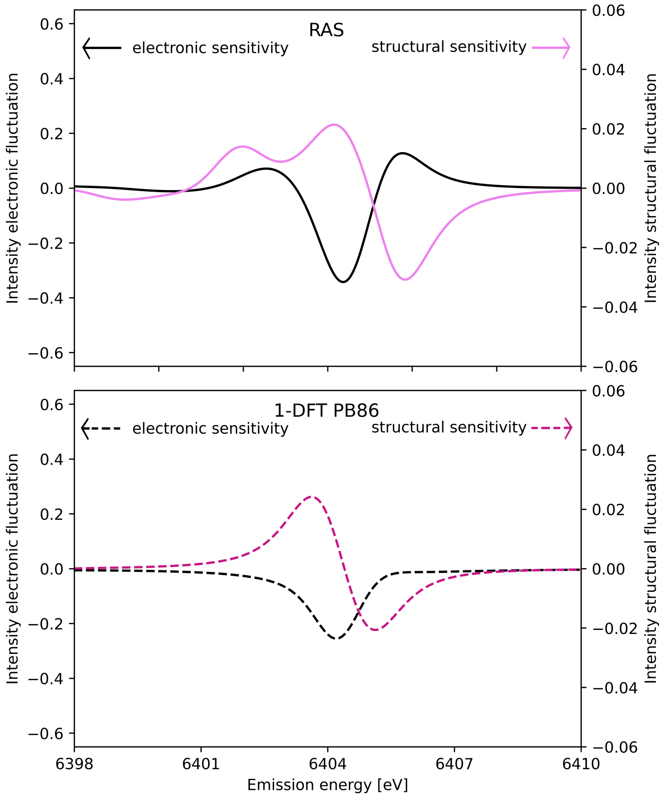

Figure S19: Kα sensitivity to electron and structural dynamics as a function of the emission energy from RAS and 1-DFT BP86. Sensitivity to electron dynamics is calculated as the difference in intensity between the ^{3}MC and GS electronic states at the ^{3}MC minimum geometry (black curve, left y-axis). Structural sensitivity represents the intensity difference between the turning points in wavepacket simulations (Fe-L distances of 2.056 Å and 2.091 Å respectively) (purple curve, right y-axis), for RAS (top) and 1-DFT PB86 (bottom). Note the different y-scales.

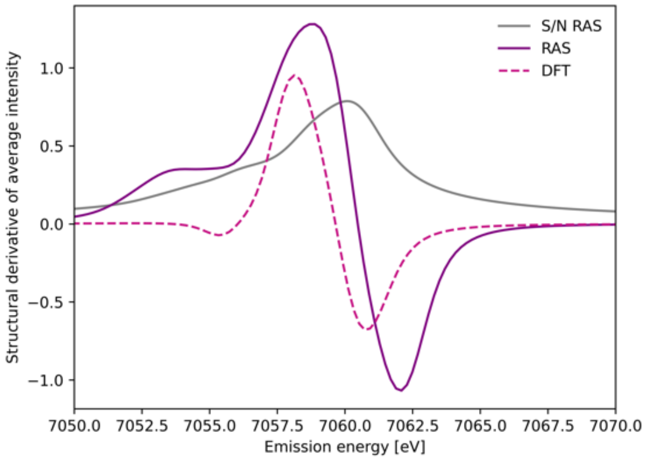

Figure S20: Kβ sensitivity to structural dynamics as a function of the normalized integrated emission energy with RAS and 1-DFT BP86 using the Total sensitivity. Signal to noise (S/N) is calculated as the square root of the ^{3}MC spectra at the ^{3}MC minimum geometry for RAS (grey curve). The normalized integrated sensitivity to structural dynamics $\delta I_{area}/\delta r$ is calculated for the ^{3}MC electronic states as the negative emission intensity variation integrated over the 7056–7058.5 eV energy range. Calculations include spectra with Fe-ligand sistances between 1.998 - 2.091 Å for RAS and 1-DFT (purple curves).

2 Additional tables

Table S1: Orbital energies and electron-nuclei interactions for an iron(II) atomic ion in the ground state (GS) and core-excited (CE) states with 1s, 2p and 3p holes.

	Orbital energy (eV)				Nuclei interaction (eV)			
Orbital	GS	CE-1s	CE-2p	CE-3p	GS	CE-1s	CE-2p	CE-3p
3s	-134.3	-160.6	-158.5	-157.2	-874.5	-895.8	-890.7	-865.8
3p	-93.8	-120.3	-117.6	-114.1	-819.3	-850.1	-842.4	-817.3
3d	-34.5	-56.3	-56.9	53.7	-679.4	-738.6	-739.4	-702.3

Table S2: Pairwise interactions (in eV) between M-shell and 1s/2p/3p electrons for an iron(II) atomic system in ground state (GS) and core-excited (CE) states.

	GS			CE-1s			CE-2p			CE-3p		
Orbitals	1s	2p	3p	1s	2p	3p	1s	2p	3p	1s	2p	3p
3s	43.4	39.6	26.1	45.2	41.3	27.5	44.8	40.8	27.1	43.8	39.9	26.5
3p	42.3	38.1	27.4	44.9	40.4	29.2	44.3	39.8	28.7	43.0	38.7	27.9
3d	33.8	33.0	24.5	37.9	36.9	27.0	37.9	36.9	26.8	35.9	35.0	25.7

References

[1] K. Kunnus, M. Vacher, T. C. B. Harlang, K. S. Kjær, K. Haldrup, E. Biasin, T. B. van Driel, M. Pápai, P. Chabera, Y. Liu, H. Tatsuno, C. Timm, E. Källman, M. Delcey, R. W. Hartsock, M. E. Reinhard, S. Koroidov, M. G. Laursen, F. B. Hansen, P. Vester, M. Christensen, L. Sandberg, Z. Németh, D. S. Szemes, É. Bajnóczi, R. Alonso-Mori, J. M. Glownia, S. Nelson, M. Sikorski, D. Sokaras, H. T. Lemke, S. E. Canton, K. B. Møller, M. M. Nielsen, G. Vankó, K. Wärnmark, V. Sundström, P. Persson, M. Lundberg, J. Uhlig and K. J. Gaffney, *Nature Communications*, 2020, **11**, 634.

[2] M. Vacher, K. Kunnus, M. G. Delcey, K. J. Gaffney and M. Lundberg, *Structural Dynamics*, 2020, **7**, 044102.
